# Comparative plastid genomics of four *Pilea* (Urticaceae) species: insight into interspecific plastid genome diversity in *Pilea*

**DOI:** 10.1186/s12870-020-02793-7

**Published:** 2021-01-07

**Authors:** Jingling Li, Jianmin Tang, Siyuan Zeng, Fang Han, Jing Yuan, Jie Yu

**Affiliations:** 1 College of Horticulture and Landscape Architecture, Southwest University, Chongqing, 400716 China; 2 College of Landscape Architecture and Life Science/Institute of Special Plants, Chongqing University of Arts and Sciences, Chongqing, 402160 China; 3 Key Laboratory of Horticulture Science for Southern Mountainous Regions, Ministry of Education, Chongqing, 400716 China

**Keywords:** *Pilea*, Plastid genome, Interspecific diversity, Hypervariable region, Phylogenetic relationship

## Abstract

**Background:**

*Pilea* is a genus of perennial herbs from the family Urticaceae, and some species are used as courtyard ornamentals or for medicinal purposes. At present, there is no information about the plastid genome of *Pilea*, which limits our understanding of this genus. Here, we report 4 plastid genomes of *Pilea* taxa (*Pilea mollis*, *Pilea glauca* ‘Greizy’, *Pilea peperomioides* and *Pilea serpyllacea* ‘Globosa’) and performed comprehensive comparative analysis.

**Results:**

The four plastid genomes all have a typical quartile structure. The lengths of the plastid genomes ranged from 150,398 bp to 152,327 bp, and each genome contained 113 unique genes, including 79 protein-coding genes, 4 rRNA genes, and 30 tRNA genes. Comparative analysis showed a rather high level of sequence divergence in the four genomes. Moreover, eight hypervariable regions were identified (*pet*N-*psb*M, *psb*Z-*trn*G-GCC, *trn*T-UGU-*trn*L-UAA, *acc*D-*psb*I, *ndh*F-*rpl*32, *rpl*32-*trn*L-UAG, *ndh*A-intron and *ycf*1), which are proposed for use as DNA barcode regions. Phylogenetic relationships based on the plastid genomes of 23 species of 14 genera of Urticaceae resulted in the placement of *Pilea* in the middle and lower part of the phylogenetic tree, with 100% bootstrap support within Urticaceae.

**Conclusion:**

Our results enrich the resources concerning plastid genomes. Comparative plastome analysis provides insight into the interspecific diversity of the plastid genome of *Pilea*. The identified hypervariable regions could be used for developing molecular markers applicable in various research areas.

**Supplementary Information:**

The online version contains supplementary material available at 10.1186/s12870-020-02793-7.

## Background

*Pilea* species are perennial herbs from the family Urticaceae and mainly distributed in tropical and subtropical regions, and some species are distributed in warm temperate regions. *Pilea* is a species-rich genus, which is the largest one in the family Urticaceae, and a relatively large genus among angiosperms [1]. The leaves of many species in *Pilea* have color spots, which can be used for garden cultivation and ornamental purposes. They are often the main plant groups in shady and humid environments of the garden landscape. On the other hand, in traditional Chinese pharmacopeia, several species are recorded as medicinal plants from which a variety of pharmacologically active substances can be extracted [2–4]. For example, *P. peperomioides* is recorded in “Dai medicine” for anti-inflammatory and detoxifying activities and is also used for erysipelas and bone setting. However, this group that has received little attention, and there are also few reports about *Pilea*. Considering that many medicinal plants are morphologically similar, especially those of these species-rich genera, accurate species identification based on molecular markers is particularly important for rational utilization of these medicinal plants.

The genus *Pilea* is also a controversial group in traditional taxonomy, and previous studies have suggested that *Sarcopilea* also belongs to this genus [5]. In addition, some new species have been reported in recent years [6, 7]. It is difficult to revise this species-rich genus with little attention from experts and scholars. Moreover, relatively little research has been reported on this genus, especially in the field of molecular biology and genomics. Though some researchers have used molecular methods to explore phylogenetic relationships within the genus *Pilea* [1] and its phylogenetic position in the family Urticaceae [5], the selected DNA fragments are one-sided and partially complete, with low bootstrap support values, which has certain limitations. It is therefore necessary for us to further study the phylogenetic relationships of *Pilea* species in Urticaceae.

The chloroplast is a kind of organelle involved in photosynthesis [8] and energy transformation in plants and algae [9, 10]. The chloroplast genome (referred to as the plastid genome or the plastome in the present text) encodes many key proteins that play essential roles in photosynthesis and other metabolic properties [11]. In previous studies, several unique characteristics of the plastome have been widely reported, such as its monophyletic inheritance [12], conserved coding region sequences [13] and genome structure [14, 15]. These reliable resources provide rich information for the study of evolution, DNA barcoding, taxonomy and phylogeny [16–18]. Although the plastid genome is highly conserved, some interesting structural variations have been observed in some taxa, such as the rare expansion of IR regions in *Strobilanthes* [15], the insertion of mitochondrial DNA in the plastome of *Anacardium* [19], and the complete or partial loss of IR regions in some legumes [16, 20, 21]. In a recent study, Wang et al. reported plastid genomes from 13 of 58 genera in Urticaceae, providing an abundance of plastid genome resources for the study of this group of plants [22]. Unfortunately, there have been no reports on the plastid genome of *Pilea* plants.

Here, we sequenced, assembled and analyzed the plastid genomes of four *Pilea* species, including a rare succulent plant of this genus (*P. serpyllacea*). As ornamental or medicinal plants, these species have great differences in morphology (especially their leaves) and are representative of the genus. Our main tasks were as follows: (1) we sequenced and assembled the plastome of four *Pilea* plant species; (2) we analyzed the structural characteristics and sequence divergence of the plastomes in *Pilea*; (3) we identified simple sequence repeats (SSRs) loci and repeat sequences for further studies on population genetic structure; (4) we inferred the phylogenetic relationships of *Pilea* in Urticaceae based on the complete plastome sequence; and (5) we identified the hypervariable regions that could be used as DNA barcodes for identification of members of this genus.

## Results

### General features of the plastid genome

Using Illumina HiSeq sequencing platforms, 5.38–5.89 Gb of clean data from each *Pilea* species were obtained, with the number of clean reads ranging from 17,935,118 to 19,627,967 (Additional File 1: Table S1). The plastome was assembled based on these data. The 4 plastomes of *Pilea* are characterized by a typical circular DNA molecule with a length ranging from 150,398 to 152,327 bp. They all have a conservative quartile structure composed of a large single copy (LSC) region (82,063 to 83,292 bp), a small single copy (SSC) region (17,487 to 18,363 bp) and a pair of inverted repeat (IR) regions (25,180 to 25,356 bp) (Table 1). The lengths of the plastomes are conserved in this genus. GC content analysis showed that the overall GC contents ranged from 36.35 to 36.69% in the 4 plastomes. Note that the GC contents in the IR regions (42.56–42.73%) are significantly higher than those in the LSC (33.87–34.36%) and SSC regions (30.01–30.81%). The raw sequencing data and the four genome sequences have been deposited into the NCBI database (accession numbers: PRJNA675740 and MT726015 to MT726018).

**Table 1:** Basic features of the 4 plastid genomes from *Pilea*

Species		*P. glauca*	*P. mollis*	*P. peperomioides*	*P. serpyllacea*
Accession number		MT726015	MT726018	MT726016	MT726017
Length (bp)	Total	151,210	150,587	152,327	150,398
	LSC	82,662	82,063	83,292	82,551
	SSC	17,836	17,864	18,363	17,487
	IR	25,356	25,330	25,336	25,180
GC content (%)	Total	36.69	36.72	36.35	36.41
	LSC	34.31	34.36	33.87	33.96
	IR	42.64	42.65	42.73	42.56
	SSC	30.81	30.76	30.01	30.23
Gene numbers	Total	133	133	133	133
	Protein-coding gene	88	88	88	88
	tRNA gene	37	37	37	37
	rRNA gene	8	8	8	8

### Genome annotation

**Fig. 1:**
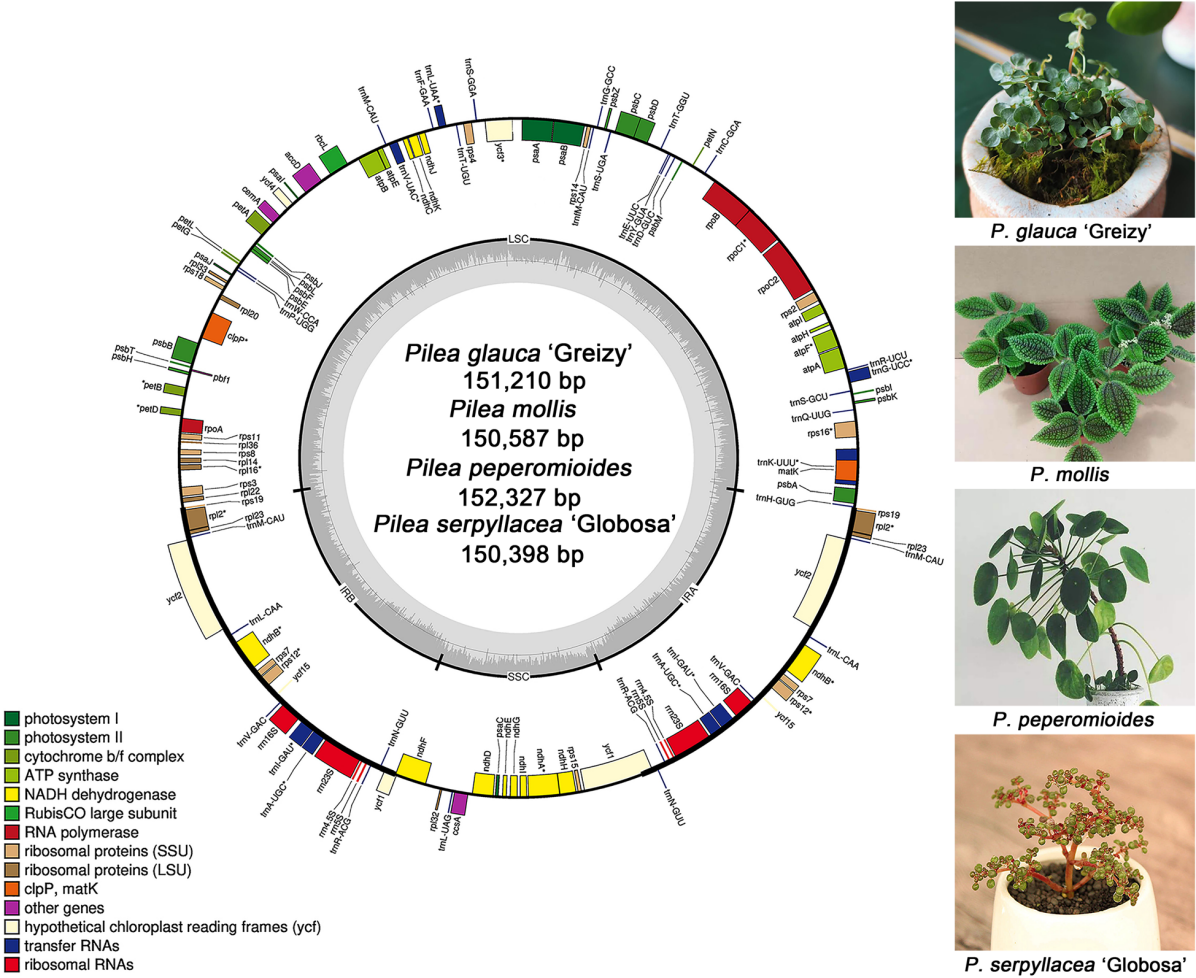
Plastid genome map of four *Pilea* species and image of the four plants. The genome has a conservative quartile structure that is composed of an LSC region, an SSC region and a pair of IR regions. The genes outside the circle are transcribed in the counterclockwise direction, and the genes inside the circle are transcribed in the clockwise direction. Different colors in genes represent different functions. The dark gray area and light gray area of the inner circle represent the ratio of GC content to AT content in the genome, respectively

The plastid genomes of the four *Pilea* species all comprise 113 unique genes, including 79 protein-coding genes, 4 rRNA genes and 30 tRNA genes (Additional File 1: Table S2). The gene order and gene numbers of these four species are highly similar, showing conserved genomic structures. Figure 1 shows the schematic diagram of the plastome of *Pilea*. Introns play a significant role in selective gene splicing [23]. Among the 79 protein-coding genes annotated, nine unique genes (*rps*16, *rpo*C1, *atp*F, *pet*B, *pet*D, *rpl*16, *rpl*2, *ndh*B, *ndh*A) contained one intron, and two unique genes (*ycf*3, *clp*P) contained two introns. Moreover, six unique tRNA genes (*trn*K-UUU, *trn*G-UCC, *trn*L-UAA*, trn*V-UAC*, trn*I-GAU, *trn*A-UGC) contain one intron. There are seven protein-coding genes, four rRNA genes, and seven tRNA genes completely duplicated in the IR regions, so they exist as two copies. The *rps*12 gene is a trans-spliced gene, and the 5′ end is located in the LSC region. However, the 3′ ends are found in the IRa and IRb regions. These results are similar to those in other species in Urticaceae [22].

### Repeat analysis

**Fig. 2:**
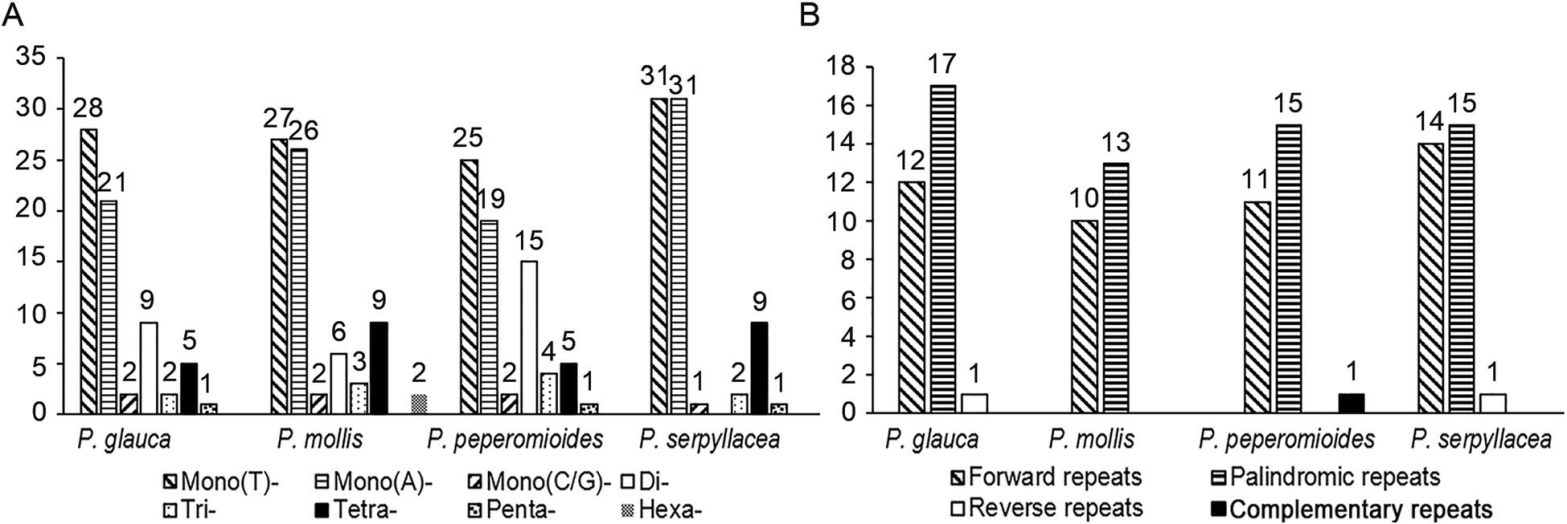
Comparison of the repeats in the plastid genomes of 4 *Pilea* species. **a.** Types and numbers of SSRs detected in the 4 plastomes; **b.** Types and numbers of interspersed repeats in the 4 plastomes

SSRs, also referred to as microsatellite sequences, provide a large amount of genetic information [24–26]. Because of its high genetic polymorphism, SSRs are often used for the development of molecular markers and play an important role in the identification of species [27, 28]. In this study, we detected 68, 75, 71, and 80 SSRs in the 4 analyzed species (Fig. 2a, Additional File 1: Table S3). Most SSRs are mononucleotide homopolymers, particularly A/T, which accounts for 70.75% of the total. Hexanucleotide repeats are detected only in *P. mollis*. These SSRs showed high polymorphism, suggesting great potential in the identification of *Pilea* species.

In the plastid genomes of *Pilea* species, we detected four types of interspersed repeats. Most of them are forward repeats and palindromic repeats (Fig. 2b). By contrast, there are only two reverse repeats and one complementary repeat. The only complementary repeats were found in *P. peperomioides*. The detailed sequences showed in Additional File 1: Table S4. Moreover, the length of these short interspersed repeats mainly ranged from 30 to 34 bp. We note one forward repeat with a length of 102 bp (detected in *P. serpyllacea*).

### Contraction and expansion analysis of IR regions

**Fig. 3:**
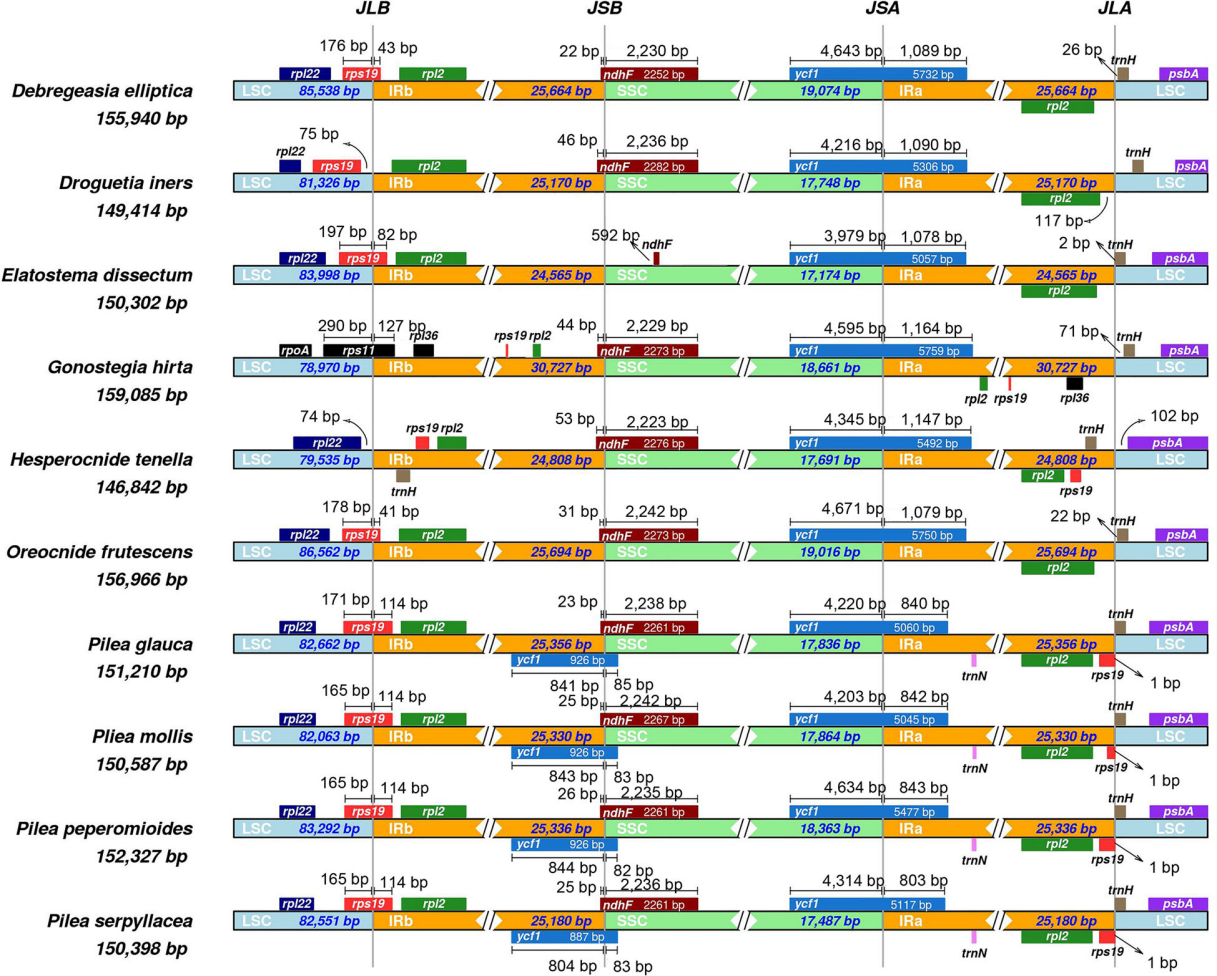
Comparison of the borders among the LSC, SSC, and IR regions of 10 analyzed species. The genes around the borders are shown above or below the mainline. JLB, JSB, JSA, and JLA represent junction sites of LSC/IRb, IRb/SSC, SSC/IRa, and IRa/LSC, respectively

The contraction and expansion of IR regions are considered to be an important reason for the length diversity in plastid genomes [29]. In addition, with the expansion/contraction of the IR regions, genes near the border have an opportunity to access IR or SC regions [30]. We retrieved the published plastomes of six species from Urticaceae and compared them with those of the four *Pilea* species. We found several genes spanning or near the boundary of the IR and SC regions. They include mainly *rps*19, *rpl*22, *rpl*2, *ycf*1, *ndh*F and *trn*H (Fig. 3). Notably, an abnormal expansion of IR regions was observed in *Gonostegia hirta*. The IR regions are more than 30,000 bp in *G. hirta*, and more genes can access the IR regions (e.g., *rpl*36 and *rps*19). However, the length of IR regions in the other nine species is approximately 25,000 bp, and the *rps*19 gene spans the LSC/IRb boundary, except in *Droguetia iners* and *Hesperocnide tenella*; the *rps*19 gene in the former is in the LSC region, while that in latter is completely in the IR region. In addition, the *trn*H gene completely accesses IR regions in *H. tenella*, obtaining two copies. It can be seen that the genomic structure, gene order and numbers of some species in Urticaceae have changed obviously.

Furthermore, the *ycf*1 gene crosses the SSC/IRa boundary, most of which is located in the SSC region. The length of the *ycf*1 gene in the four *Pilea* species varies widely, indicating the possibility of sequence differences. Surprisingly, we annotated two copies of *ycf*1 in the four *Pilea* plants; they cross the IRb/SSC boundary and are not annotated in other species. Sequence alignment found that the two copies of *ycf*1 exist in other taxa, indicating that the previous annotation is imperfect, although one of the two copies is a fragment of *ycf*1 and is generally considered to be a pseudogene. Interestingly, a small fraction of the *ndh*F gene (less than 100 bp) crosses the IRb/SSC regions, which means that the first copy of *ycf*1 has an overlap with *ndh*F in *Pilea* species. The overlapping areas are 108 bp in length.

### Genomic divergence

**Fig. 4:**
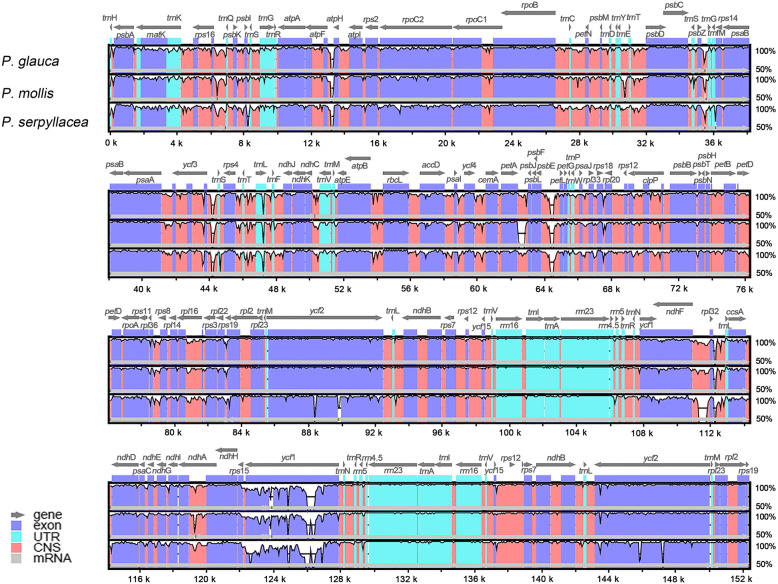
Comparison of the plastomes in the 4 *Pilea* species by using mVISTA. The genes are represented as gray arrows on the top of the alignments. The different regions are labeled with different colors. The pink regions are “conserved noncoding sequences” (CNS), the dark blue regions are exons, and the light-blue regions are tRNAs or rRNAs. The percentages 50 and 100% refer to the similarity among sequences. The gray arrows above the aligned sequences represent genes and their orientation

To evaluate the genomic divergence, sequence identity analysis based on mVISTA [31] was performed among the 4 *Pilea* species, with the reference being the plastome of *P. peperomioides*. We observed varying degrees of sequence divergence, especially in the LSC and SSC regions. In contrast, the IR regions were more conserved. Most of these highly variable regions were observed in conserved noncoding sequences (CNS) (Fig. 4). However, the regions with the greatest sequence divergence were found in protein-coding regions, in which the gene *ycf*1 is present. The coding regions of *ycf*1 in the four *Pilea* species showed significant differences, and the similarity was even less than 50% for some fragments. Overall, the analyzed genomic sequences showed rather high levels of sequence divergence throughout the genus *Pilea*.

**Fig. 5:**
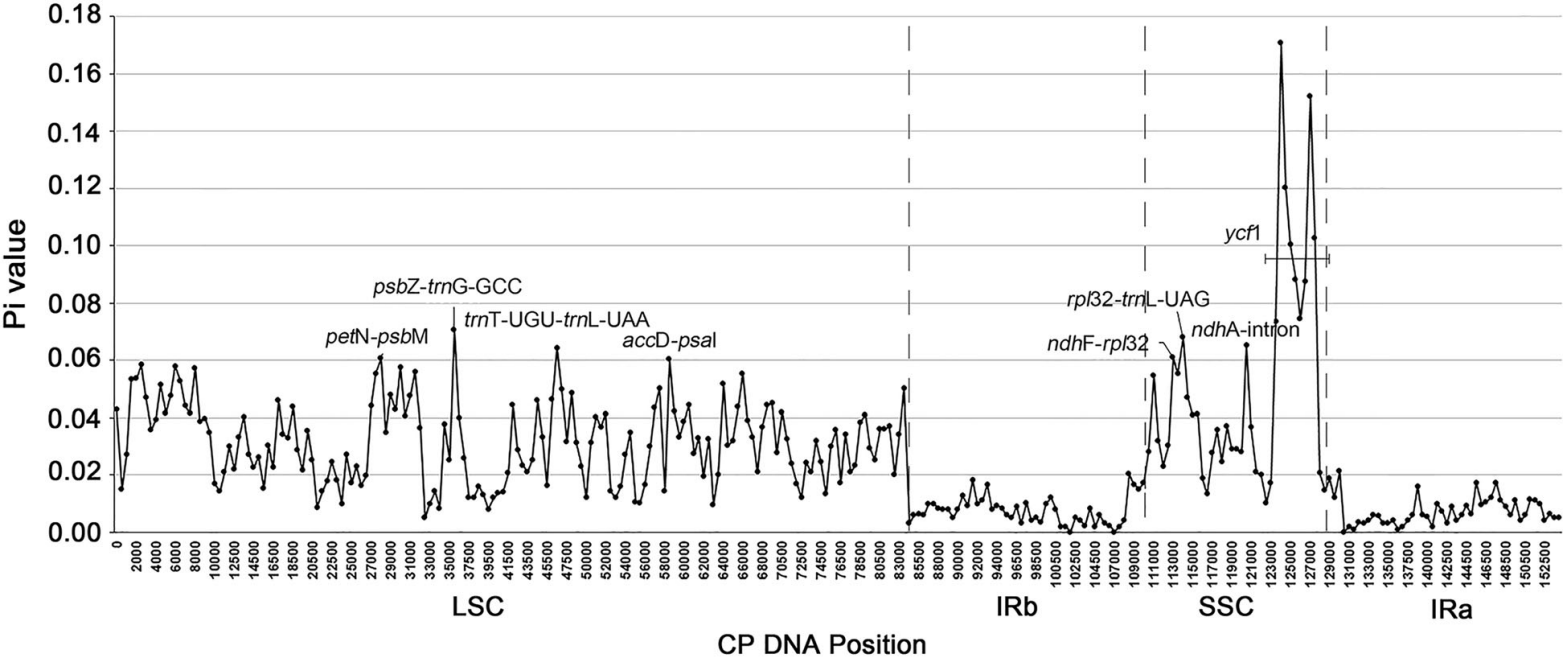
Nucleotide diversity (Pi) of plastomes among the 4 *Pilea* species. Each black dot represents the nucleotide diversity per 500 bp. Seven intergenic regions (*pet*N-*psb*M, 0.06067; *psb*Z-*trn*G-GCC, 0.07067; *trn*T-UGU-*trn*L-UAA, 0.06433; *acc*D-*psb*I, 0.06003; *ndh*F-*rpl*32, 0.06100; *rpl*32-*trn*L-UAG, 0.06800; *ndh*A-intron, 0.06533) and one protein-coding region (*ycf*1, 0.07367–0.17067) had Pi values greater than 0.06

To quantify the levels of DNA polymorphism, the 4 genomes were aligned and analyzed by using DnaSP v6.0 [32]. We detected 8 hypervariable regions, with Pi values exceeding 0.06 (Fig. 5), *pet*N-*psb*M (Pi = 0.06067); *psb*Z-*trn*G-GCC (Pi = 0.07067); *trn*T-UGU-*trn*L-UAA (Pi = 0.06433); *acc*D-*psb*I (Pi = 0.06003); *ndh*F-*rpl*32 (Pi = 0.06100); *rpl*32-*trn*L-UAG (Pi = 0.06800); *ndh*A-intron (Pi = 0.06533), and most regions of the gene *ycf*1 (Pi values ranging from 0.07367 to 0.17067). The Pi values are listed in parentheses. Notably, most regions of the plastome sequences had Pi values greater than 0.02 (except for IR regions), exhibiting abundant polymorphism of the plastid genome in *Pilea*.

### Nucleotide variations in protein-coding genes

**Fig. 6:**
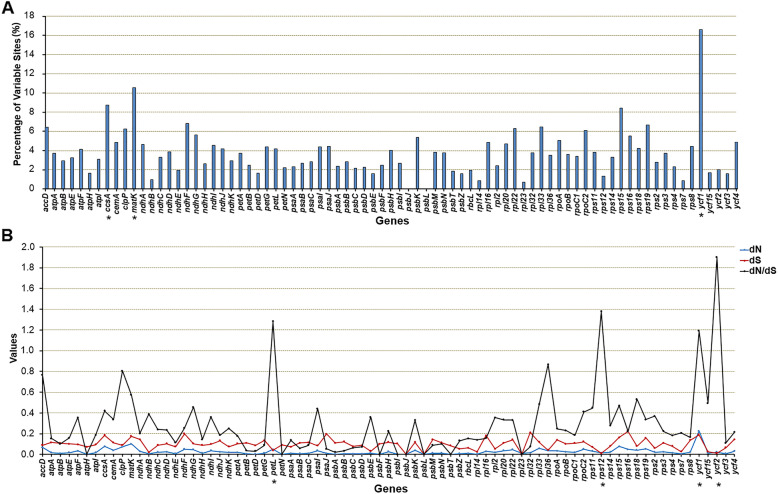
Sequence polymorphism among 79 shared plastid genes of 4 *Pilea* species. b. Percentages of variable sites in 79 shared protein-coding genes. We used MEGA v6.0 to calculate the percentages of variable sites. The three genes with the highest mutation rates are labeled with an *: *ycf*1 (16.62%), *mat*K (10.54%) and *ccs*A (8.74%). **b**. Estimations of nonsynonymous (dN) and synonymous (dS) substitution rates and the dN/dS of 79 shared protein-coding genes. The four genes with the highest dN/dS values are labeled with an *

The protein-coding regions are highly conserved in plastid genomes [33]. We analyzed the protein-coding sequences of 79 identified unique orthologous genes in 4 *Pilea* taxa. Surprisingly, these protein-coding genes also showed high levels of variation (Fig. 6a, Additional File 1: Table S5). Of the 79 shared genes, 63 had a mutation rate of more than 2%, and 30 had a mutation rate of more than 4%. The gene with the highest mutation rates was *ycf*1 (16.62%), followed by *mat*K (10.54%), *ccs*A (8.74%) and *rps*15 (8.42%). Only two genes (*psb*J and *psb*L) showed extreme conservation without any variable sites. Moreover, we observed a total of 11 genes (*ycf*1, *ndh*F, *rps*19, *acc*D, *rpo*C2, *rps*16, *rpo*A, *rpl*20, *ndh*D, *rpo*C1 and *ycf*2) with InDels in nucleotide sequences by using DnaSP v6.0 [32]. Among these, *ycf*1 had 35 InDels, followed by *ycf*2 (9), *acc*D (4) and *rpo*C2 (3). Considering that the protein-coding regions are highly conserved, protein-coding sequences with high nucleotide mutation rates are usually infrequent in the same genus, and these results showed interspecific diversity within the plastid genome of *Pilea*.

In this study, synonymous (dS) and nonsynonymous (dN) substitution rates, along with dN/dS, were estimated for the 79 shared genes in parallel by using PAML v4.9 [34]. Among the 79 genes, *ycf*1, *mat*K, *ccs*A and *rps*15 had relatively high dN values, and *rps*16, *rpl*32, *ndh*F and *psa*J had relatively high dS values (Fig. 6b, Additional file 1: Table S6). Most genes exhibited considerably low dN/dS values (less than 0.6), implying that most of the protein-coding genes were under purifying selection during evolution. However, the dN/dS ratio of three genes (*rpl*36, *clp*P and *acc*D) was between 0.6 and 1.0. Moreover, the dN/dS ratio was greater than 1.0 for *pet*L, *rps*12, *ycf*1 and *ycf*2, indicating that they were under positive selection during evolution. These results clearly indicated that the plastid genes in the different species of *Pilea* may have been subjected to different selection pressures.

### Phylogenetic analysis

**Fig. 7:**
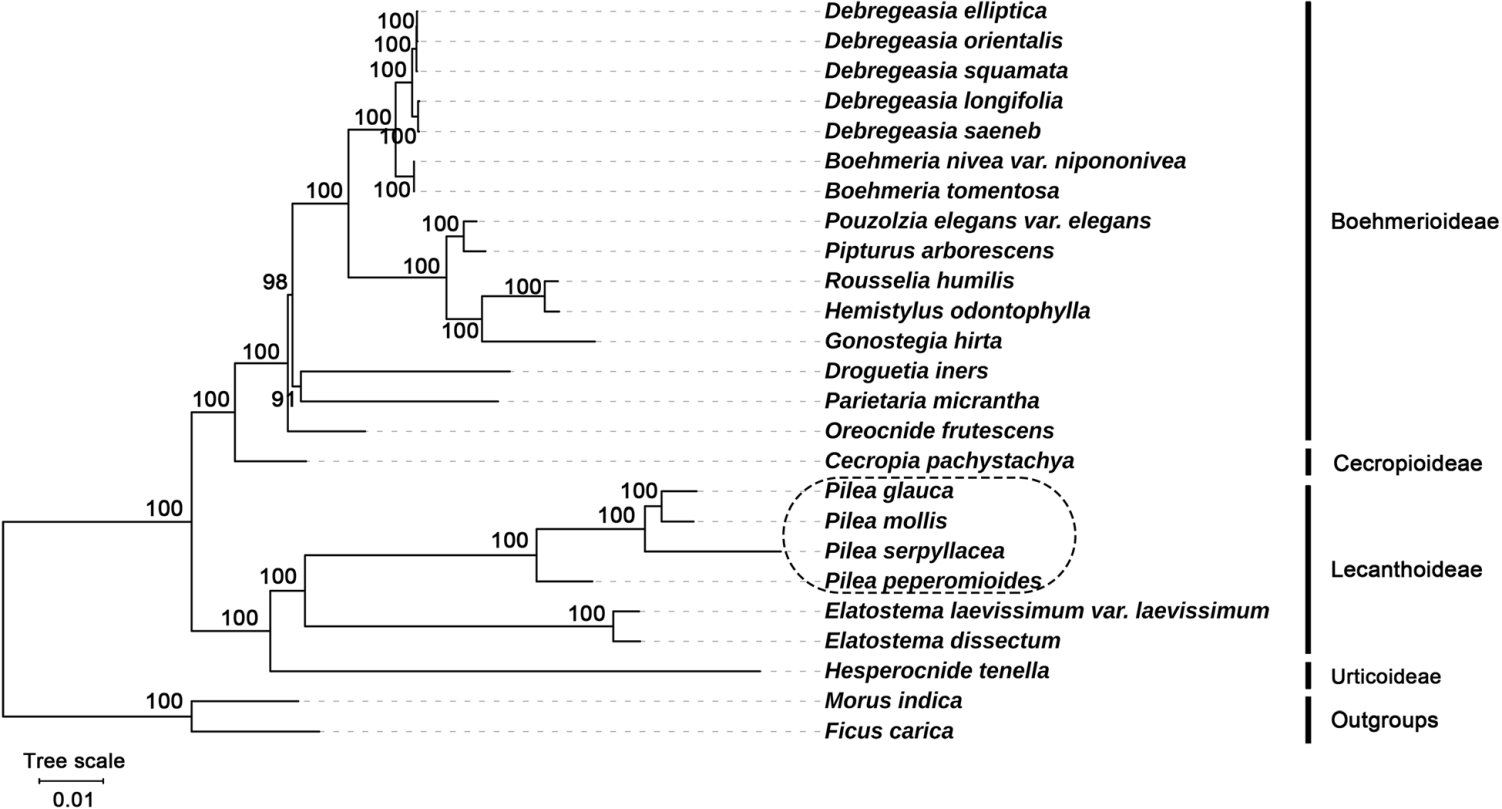
Phylogenetic relationships of species from Urticaceae inferred using the maximum likelihood (ML) method. The phylogenetic tree was constructed using the complete plastome sequences among the 25 plastid genomes. The number at the bottom of the scale, 0.01, means that the length of the branch represents the replacement frequency of bases at each site of the genome at 0.01. The bootstrap values were calculated from 1000 replicates. Two taxa from Moraceae, nam

In this study, we constructed maximum likelihood (ML) trees by using the complete plastome sequences as data sets (detailed materials are shown in Additional File 1: Table S7). The phylogenetic tree has high bootstrap support in all nodes, showing the reliability of the phylogeny recovered (Fig. 7).

Our phylogenetic tree displayed two clades clearly and then further diversified into four subclades with 100% bootstrap support (ML). These four subclades correspond to four subfamilies: Boehmerioideae, Cecropioideae, Lecanthoideae and Urticoideae. This is consistent with the traditional classification [5]. All 4 *Pilea* species clustered together (all nodes have BS = 100 for the ML method) and formed a monophyletic group that is a sister group to *Elatostema*. They all belong to the subfamily Lecanthoideae.

## Discussion

### Conserved genome structure and gene content

In our study, we reported four plastid genomes of *Pilea* taxa for the first time. Our assembly results showed that the lengths of the 4 plastid genomes ranged from 150,398 bp to 152,327 bp and that they have a typical tetrad structure. In terms of structure, these results are similar to those of most Urticaceae plants [35, 36]. In this study, the longest and shortest plastid genomes in Urticaceae were 159,085 bp (*Gonostegia hirta*) and 146,842 bp (*Hesperocnide tenella*), respectively. This suggests that the plastid genomes of Urticaceae may have undergone different evolutionary processes. Among our four *Pilea* taxa, the longest genome sequence was that of *P. peperomioides* (152,327 bp) and the shortest was that of *P. serpyllacea* (150,398 bp), and we did not detect gene gain or loss, suggesting that the plastomes are still relatively conserved in *Pilea*.

Moreover, we detected SSRs and repeat sequences in the four plastid genomes. Of the 294 total SSRs, 215 are mononucleotide repeats, accounting for the majority of all SSRs (73.13%). These mononucleotide repeats are mainly A/T repeats, and they have a significant impact on the overall G/C content of the genomes [37, 38]. These SSR sequences are often composed of simple repeating units such as polyadenine (Poly-A) or polythymine (Poly-T) repeats. With length polymorphisms in different species, they are often used as molecular markers. These abundant SSR loci in plastomes have been applied in species identification [16, 39]. Interspersed repeats are thought to be essential for promoting plastome rearrangements [40, 41]. We note that there is one forward repeat with a length of 102 bp in *P. serpyllacea*, and this is effective for increasing the length of the plastome. Whether these repeats caused the rearrangement of the plastomes of *Pilea* species is an interesting question.

Variation in IR regions is a common phenomenon in angiosperms. Compared with the overall absence of one IR region [42–44], the expansion/contraction of IR regions is more common in angiosperms [45, 46]. By comparative analysis, we found that *G. hirta* has significantly expanded IR regions, which also led to an increase in the overall length of the plastome. In our tested four *Pilea* species, the length of the IR regions ranged from 25,180 bp to 25,356 bp, showing no significant difference. As far as the boundary regions of IR/SC are concerned, the position of genes near the boundary in the four *Pilea* species is similar to that in most angiosperms. This indicates that the *Pilea* species did not experience significant expansion/contraction in the IR regions. However, we observed that the overlap of the *ycf*1 and SSC regions (4634 bp) in *P. peperomioides* was longer than that in the other three species (4203 bp-4314 bp), and the overlap with IRa was similar (803 bp–843 bp). This suggests that there is a significant difference in *ycf*1 gene sequences. In addition, the first copy of *ycf*1 overlaps with *ndh*F in *Pilea* taxa, and this result is also observed in *Arabidopsis*; the overlaps are approximately 30 bp [47]. Whether these overlaps affect the transcription or translation of these proteins is also an interesting subject.

### Sequence divergence reveals the interspecific diversity of plastid genomes in *Pilea*

In our comparative plastid genomics analysis, we first compared the whole plastid genomes based on mVISTA. Specifically, we also calculated the percentage of variable sites and estimated the ratios of dN/dS among 79 orthologous protein-coding genes. Like in most angiosperms, the noncoding regions of plastomes in *Pilea* showed higher polymorphism than did the coding regions. Surprisingly, we also found rather high levels of sequence differences in the coding regions of *Pilea* taxa. Of the 79 orthologous genes identified, 63 had a mutation rate of more than 2%, and 30 had a mutation rate of more than 4%. This is rare in other genera because usually only the *ycf*1 gene has a high mutation rate [48]. The mutation rate of the *ycf*1 gene in the four *Pilea* species is an astonishing 16.62%. Additionally, a total of 35 InDels were detected, including a large fragment insertion in *P. peperomioides* (177 bp, data not shown). These InDels caused an increase in the length of the *ycf*1 gene in *P. peperomioides*. In addition, unusually high nucleotide mutation rates were also observed in *mat*K, *ccs*A and other genes.

In general, dN changes are subject to bidirectional effects of varied mutation rates and selective constraints. A ratio of dN/dS greater than 1 is thought to be a sign that the gene has experienced selection pressure. In our study, the dN/dS ratios indicate that four genes (*pet*L, *rps*12, *ycf*1 and *ycf*2) may have undergone positive selection in *Pilea*. The rapid evolution of protein-coding genes is closely related to the adaptive evolution of species [49, 50], indicating that *Pilea* species may have experienced a rapid evolutionary process, resulting in a species-rich genus.

### Eight hypervariable regions could be used as potential DNA barcodes

Moreover, we used DnaSP v6.0 to quantify DNA sequence polymorphisms by conducting a sliding window analysis (window length, 500 bp; step size, 500 bp). Similar to the results of mVISTA, most regions except IR regions have high Pi values, which means that several regions have potential for the development of molecular markers. We recommend eight hypervariable regions, *pet*N-*psb*M (Pi = 0.06067); *psb*Z-*trn*G-GCC (Pi = 0.07067); *trn*T-UGU-*trn*L-UAA (Pi = 0.06433); *acc*D-*psb*I (Pi = 0.06003); *ndh*F-*rpl*32 (Pi = 0.06100); *rpl*32-*trn*L-UAG (Pi = 0.06800); *ndh*A-intron (Pi = 0.06533) and almost the entire *ycf*1 gene (Pi values ranging from 0.07367 to 0.17067), as potential molecular markers for *Pilea* taxa. In particular, the gene *ycf*1, with a large number of InDels, can be used as a specific molecular marker, which is of great significance for us to correctly identify and rationally utilize medicinal taxa from this genus.

### Phylogenetic analysis of *Pilea* based on the plastid genome

Compared to nuclear and mitochondrial genomes, plastid genomes are highly conserved, and they have been widely used in phylogenetic and evolutionary studies [51–53]. With the development of high-throughput sequencing technology, the chloroplast genome sequence plays an important role in species identification as a super barcode [54, 55].

The phylogenetic relationships of *Pilea* in Urticaceae were analyzed based on the complete plastome sequences. In a one-sided analysis based on plastid genomes, *Pilea* and *Elatostema* were found to be sister groups to each other, both belonging to the subfamily Lecanthoideae. This is consistent with the results of traditional classification studies [5]. However, due to the maternal inheritance of the plastid genome [56], these results are limited. Accurate phylogenetic relationships still require a comprehensive analysis of nuclear and organellar genes [57]. Furthermore, only 14 of the 58 genera of Urticaceae have been sequenced to date. More genome sequencing is needed in the future to determine the relationships among *Pilea* and other species from the family Urticaceae.

## Conclusions

In this study, four plastid genomes of *Pilea* were sequenced and assembled for the first time in this genus. These 4 plastomes have similar structural characteristics and a typical quartile structure similar to that in most angiosperms. Unusually, the sequences of these 4 plastomes, including the relatively conserved protein-coding regions, have rather high levels of variability, which provides insight into the interspecific diversity of the plastid genome of *Pilea*. In addition, eight hypervariable regions were identified, which could be used as molecular markers for the identification of this genus. Our results enrich the data on the plastid genomes of Urticaceae and provide the basis for the phylogenetic reconstruction of *Pilea*.

## Methods

### Plant material, DNA extraction and sequencing

Fresh leaves of four *Pilea* species were collected from the local flower market of Guangzhou, Kunming and Suqian. They were identified by Professor Jie Yu. These species are cultivated as ornamental plants, and no permission is required to collect these samples. Our experimental research, including the collection of plant materials, are complies with institutional, national or international guidelines. All the samples were deposited in the Herbarium of Southwest University, Chongqing, China (voucher code: UP200602 to UP200605). The detailed information for the plant samples shown in Additional File 1: Table S8. Total genomic DNA was extracted by using the CTAB method [58]. The DNA library with an insert size of 350 bp was constructed using a NEBNext® library construction kit and sequenced by using the HiSeq Xten PE150 sequencing platform. Sequencing produced a total of 5.4–5.9 Gb of raw data per species. Clean data were obtained by removing low-quality sequences, including sequences with a quality value of Q < 19 that accounted for more than 50% of the total bases and sequences in which more than 5% bases were “N”.

### Genome assembly and annotation

De novo genome assembly from the clean data was accomplished utilizing NOVOPlasty v2.7.2 [59], with a k-mer length of 39 bp and a sequence fragment of the *rbc*L gene from maize as the seed sequence. The correctness of the assembly was confirmed by using Bowtie2 (v2. 0.1) [60] to manually edit and map all the raw reads to the assembled genome sequence under the default settings. The plastid genome was annotated initially by using CPGAVAS2 [61] with a reference genome (*Elatostema dissectum*, GenBank: NC_047192.1). GeSeq was then used to confirm the annotation results [62]. Furthermore, the annotations with problems were manually edited by using Apollo [63], and genome maps were drawn by OGDRAW [64]. The raw sequencing data and the four genome sequences have been deposited in GenBank (accession numbers: PRJNA675740; MT726015, MT726016, MT726017 and MT726018).

### Repeats and SSR analysis

The GC content was determined by using the cusp program provided by EMBOSS (v6.3.1) [65]. The simple sequence repeats (SSRs) were identified using the online website MISA (https://webblast.ipk-gatersleben.de/misa/), including mono-, di-, tri-, tetra-, penta-, and hexanucleotides with minimum numbers of 10, 5, 4, 3, 3, and 3, respectively [66]. Additionally, REPuter (https://bibiserv.cebitec.uni-bielefeld.de/reputer/) was used to calculate palindromic repeats, forward repeats, reverse repeats, and complementary repeats with the following settings: hamming distance of three and minimal repeat size of 30 bp [67].

### Genome comparison

The plastomes of the 4 *Pilea* species were compared by using the shuffle-LAGAN mode in mVISTA [68, 69] to identify interspecific variations (http://genome.lbl.gov/vista/mvista/submit.shtml). A total of 79 orthologous genes among the 4 species were identified and extracted by using PhyloSuite [70]. The corresponding nucleotide sequences were aligned by using MAFFT (v 7.450) [71] implemented in PhyloSuite. We used MEGA v6.0 [72] to calculate the percentage of variable sites in the protein-coding genes. We also conducted a sliding window analysis (window length: 500 bp, step size: 500 bp) by using DnaSP v6.0 [32] to calculate the nucleotide polymorphism (Pi) among the 4 species. Finally, IRscope (https://irscope.shinyapps.io/irapp/) was used to visualize the IR boundaries in these genomes [73].

### Analysis of the nucleotide substitution rate

The protein-coding sequences in the previous step were processed in parallel. We used the CODEML module in PAML v.4.9 [34] to estimate the rates of nucleotide substitution, including dN, dS, and the ratio of dN to dS. The detailed parameters were as follows: CodonFreq = 2 (F3 × 4 model); model = 0 (allowing a single dN/dS value to vary among branches); cleandata = 1 (removing sites with ambiguous data); and other parameters in the CODEML control file set to the default settings. A phylogenetic tree of each gene was generated by using the maximum likelihood (ML) method implemented in RAxML v8.2.4 [74].

### Phylogenetic analysis

The plastid genomes of 19 species belonging to the family Urticaceae were downloaded from GenBank (NCBI, https://www.ncbi.nlm.nih.gov/). These species belong to 4 subfamilies (Additional File 1: Table S7). Two species from Moraceae (*Morus indica* and *Ficus carica*) were used as outgroups. The complete plastome sequences were aligned by using MAFFT (https://mafft.cbrc.jp/alignment/server/) online version 7.471 [71]. These aligned sequences were used to construct the phylogenetic trees by using the maximum likelihood (ML) method implemented in RAxML v8.2.4 [74]. The parameters were “raxmlHPC-PTHREADS-SSE3 -f a -N 1000 -m GTRGAMMA -x 551314260 -p 551314260”. The bootstrap analysis was performed with 1000 replicates.

## Supplementary Information

**Additional file 1: Table S1.** Summary of sequencing data quality. **Table S2.** Gene composition in the plastid genomes of *Pilea*. **Table S3.** Statistics on simple sequence repeats (SSRs) in the 4 plastid genomes. **Table S4.** Repeats (> = 30 bp) identified in the four *Pilea* species. **Table S5**. Percentages of variable sites and Indels in orthologous genes among the 4 *Pilea* species. **Table S6**. The dS, dN and dN/dS values in 79 shared genes among 4 *Pilea* species. **Table S7**. List of plastid genomes used for phylogenetic analysis. Table S8. Summary information of the plant samples.

## Data Availability

The raw sequencing data generated in this study and the four plastid genome sequences were deposited in NCBI (https://www.ncbi.nlm.nih.gov/) with accession number: PRJNA675740, MT726015, MT726016, MT726017 and MT726018. All the samples are saved at the Herbarium of Southwest University, Chongqing, China. All other data and material generated in this manuscript are available from the corresponding author upon reasonable request.

## Abbreviations

SSR: Simple sequence repeat
CNS: Conserved Non-Coding Sequences
IRs: Inverted repeats
LSC: Large single-copy
SSC: Small single-copy
ML: Maximum-likelihood
BS: Branch support
PolyA: Polyadenine
PolyT: Polythymine
dS: Synonymous substitution rates
dN: Nonsynonymous substitution rates
DnaSP: DNA Sequences Polymorphism
CTAB: Cetyl trimethylammonium bromide
NCBI: National Center for Biotechnology Information
Pi: Nucleotide diversity/polymorphism
