# Supplementary material for: Comparative plastid genomics of four Pilea (Urticaceae) species: insight into interspecific plastid genome diversity in Pilea
Source: BMC Plant Biol. 2021 Jan 7;21:25. doi: 10.1186/s12870-020-02793-7 (PMC7792329; doi:10.1186/s12870-020-02793-7)
Supplement: Supplementary file 1 — Additional file 1: Table S1. Summary of sequencing data quality. Table S2. Gene composition in the plastid genomes of Pilea. Table S3. Statistics on simple sequence repeats (SSRs) in the 4 plastid genomes. Table S4. Repeats (> = 30 bp) identified in the four Pilea species. Table S5. Percentages of variable sites and Indels in orthologous genes among the 4 Pilea species. Table S6. The dS, dN and dN/dS values in 79 shared genes among 4 Pilea species. Table S7. List of plastid genomes used for phylogenetic analysis. Table S8. Summary information of the plant samples. [file 12870_2020_2793_MOESM1_ESM.zip › Table S1.docx]

**Table S1.** Summary of sequencing data quality.

| Sample | *Pilea glauca* | *Pilea peperomioides* | *Pilea serpyllacea* | *Pilea mollis* |
| --- | --- | --- | --- | --- |
| Raw Reads | 17,988,506 | 19,321,323 | 17,999,002 | 19,676,716 |
| Clean Reads | 17,935,118 | 19,226,971 | 17,952,829 | 19,627,967 |
| Raw Base (Gb) | 5.4 | 5.8 | 5.4 | 5.9 |
| Clean Base (Gb) | 5.38 | 5.77 | 5.39 | 5.89 |
| Effective Rate (%) | 99.7 | 99.51 | 99.74 | 99.75 |
| Error Rate (%) | 0.03 | 0.03 | 0.03 | 0.03 |
| Q20 (%) | 96.72 | 97.00 | 96.92 | 97.06 |
| Q30 (%) | 91.36 | 91.79 | 91.63 | 91.75 |

Note. Error Rate (%): mean error rate of base sequencing, Q20, Q30: The percentage of bases whose Phred value is greater than 20 or 30 in the total base.
