# Supplementary material for: Comparative plastid genomics of four Pilea (Urticaceae) species: insight into interspecific plastid genome diversity in Pilea
Source: BMC Plant Biol. 2021 Jan 7;21:25. doi: 10.1186/s12870-020-02793-7 (PMC7792329; doi:10.1186/s12870-020-02793-7)
Supplement: Supplementary file 1 — Additional file 1: Table S1. Summary of sequencing data quality. Table S2. Gene composition in the plastid genomes of Pilea. Table S3. Statistics on simple sequence repeats (SSRs) in the 4 plastid genomes. Table S4. Repeats (> = 30 bp) identified in the four Pilea species. Table S5. Percentages of variable sites and Indels in orthologous genes among the 4 Pilea species. Table S6. The dS, dN and dN/dS values in 79 shared genes among 4 Pilea species. Table S7. List of plastid genomes used for phylogenetic analysis. Table S8. Summary information of the plant samples. [file 12870_2020_2793_MOESM1_ESM.zip › Table S2.docx]

**Table S2.** Gene composition in the plastid genomes of *Pilea*.

| **Category of Genes** | **Group of Genes** | **Name of Genes** |
| --- | --- | --- |
| Self-replication | Ribosomal RNA | *rrn*16S (x2), *rrn*23S (x2), *rrn*5S (x2), *rrn4.*5S (x2) |
|  | Transfer RNA | *trn*A-UGC (x2)**, trn*C-GCA*, trn*D-GUC*, trn*E-UUC*, trn*F-GAA*, trn*fM-CAU*, trn*G-GCC*, trn*G-UCC**, trn*H-GUG*, trn*I-CAU*, trn*I-GAU (x2)**, trn*K-UUU**, trn*L-CAA (x2)*, trn*L-UAA**, trn*L-UAG (x2)*, trn*M-CAU (x2)*, trn*N-GUU (x2)*, trn*P-UGG*, trn*Q-UUG*, trn*R-ACG*, trn*R-UCU*, trn*S-GCU*, trn*S-GGA*, trn*S-UGA*, trn*T-GGU*, trn*T-UGU*, trn*V-GAC (x2)*, trn*V-UAC**, trn*W-CCA*, trn*Y-GUA |
|  |  |  |
|  |  |  |
|  |  |  |
|  |  |  |
|  |  |  |
|  |  |  |
|  |  |  |
|  | Large subunit of ribosome | *rpl*14, *rpl*16*, *rpl*2 (x2)*, *rpl*20, *rpl*22, *rpl*23 (x2), *rpl*32, *rpl*33, *rpl*36 |
|  | DNA dependent RNA polymerase | *rpo*A, *rpo*B, *rpo*C1*, *rpo*C2 |
|  | Small subunit of ribosome | *rps*11, *rps*12 (x2)*, *rps*14, *rps*15, *rps*16*, *rps*18, *rps*19, *rps*2, *rps*3, *rps*4, *rps*7(x2), *rps*8 |
| Photosynthesis | Subunits of ATP synthase | *atp*A, *atp*B, *atp*E, *atp*F*, *atp*H, *atp*I |
|  | Subunits of photosystem II | *psb*A, *psb*B, *psb*C, *psb*D, *psb*E, *psb*F, *psb*H, *psb*I, *psb*J, *psb*K, *psb*L, *psb*M, *psb*N, *psb*T, *psb*Z, *ycf*3** |
|  |  |  |
|  | Subunits of NADH-dehydrogenase | *ndh*A*, *ndh*B (x2)*, *ndh*C, *ndh*D, *ndh*E, *ndh*F, *ndh*G, *ndh*H, *ndh*I, *ndh*J, *ndh*K |
|  |  |  |
|  | Subunits of cytochrome b/f complex | *pet*A, *pet*B*, *pet*D*, *pet*G, *pet*L, *pet*N |
|  | Subunits of photosystem I | *psa*A, *psa*B, *psa*C, *psa*I, *psa*J |
|  | Subunit of rubisco | *rbc*L |
| Other Genes | Subunit of Acetyl-CoA-carboxylase | *acc*D |
|  | c-type cytochrom synthesis gene | *ccs*A |
|  | Envelop membrane protein | *cem*A |
|  | Protease | *clp*P** |
|  | Maturase | *mat*K |
| Unknown | Conserves open reading frames | *ycf*1, *ycf*15 (x2), *ycf*2 (x2), *ycf*4 |
|  | Gene Fragments (pseudogene) | *ycf*1, *rps*19 |

Note. (x2) indicates that the gene located in the IRs and thus had two complete copies, * and ** indicate that genes containing one/ two introns.
