# Supplementary material for: Comparative plastid genomics of four Pilea (Urticaceae) species: insight into interspecific plastid genome diversity in Pilea
Source: BMC Plant Biol. 2021 Jan 7;21:25. doi: 10.1186/s12870-020-02793-7 (PMC7792329; doi:10.1186/s12870-020-02793-7)
Supplement: Supplementary file 1 — Additional file 1: Table S1. Summary of sequencing data quality. Table S2. Gene composition in the plastid genomes of Pilea. Table S3. Statistics on simple sequence repeats (SSRs) in the 4 plastid genomes. Table S4. Repeats (> = 30 bp) identified in the four Pilea species. Table S5. Percentages of variable sites and Indels in orthologous genes among the 4 Pilea species. Table S6. The dS, dN and dN/dS values in 79 shared genes among 4 Pilea species. Table S7. List of plastid genomes used for phylogenetic analysis. Table S8. Summary information of the plant samples. [file 12870_2020_2793_MOESM1_ESM.zip › Table S3.docx]

**Table S3.** Statistics on simple sequence repeats (SSRs) in the 4 plastid genomes.

| Species | SSR nr. | SSR type | SSR | Size | Start | End |
| --- | --- | --- | --- | --- | --- | --- |
| *Pilea glauca* | 1 | p1 | (A)11 | 11 | 110 | 120 |
| *Pilea glauca* | 2 | p1 | (A)18 | 18 | 3622 | 3639 |
| *Pilea glauca* | 3 | p1 | (A)13 | 13 | 4388 | 4400 |
| *Pilea glauca* | 4 | p4 | (ATCA)3 | 12 | 4708 | 4719 |
| *Pilea glauca* | 5 | p4 | (TTAA)3 | 12 | 4968 | 4979 |
| *Pilea glauca* | 6 | p1 | (A)13 | 13 | 5984 | 5996 |
| *Pilea glauca* | 7 | p2 | (AT)5 | 10 | 6411 | 6420 |
| *Pilea glauca* | 8 | p1 | (A)11 | 11 | 8455 | 8465 |
| *Pilea glauca* | 9 | p1 | (T)11 | 11 | 12291 | 12301 |
| *Pilea glauca* | 10 | p1 | (A)11 | 11 | 12453 | 12463 |
| *Pilea glauca* | 11 | p3 | (TTA)4 | 12 | 15126 | 15137 |
| *Pilea glauca* | 12 | p1 | (T)10 | 10 | 17964 | 17973 |
| *Pilea glauca* | 13 | p1 | (T)10 | 14 | 18070 | 18083 |
| *Pilea glauca* | 14 | p2 | (TA)5 | 10 | 19458 | 19467 |
| *Pilea glauca* | 15 | p1 | (T)10 | 10 | 25835 | 25844 |
| *Pilea glauca* | 16 | p2 | (AT)5 | 10 | 28904 | 28913 |
| *Pilea glauca* | 17 | p1 | (T)13 | 13 | 30514 | 30526 |
| *Pilea glauca* | 18 | p1 | (T)10 | 10 | 31421 | 31430 |
| *Pilea glauca* | 19 | p1 | (G)10 | 10 | 33572 | 33581 |
| *Pilea glauca* | 20 | p1 | (A)14 | 14 | 34625 | 34638 |
| *Pilea glauca* | 21 | p1 | (C)11 | 11 | 35831 | 35841 |
| *Pilea glauca* | 22 | p1 | (T)10 | 10 | 41995 | 42004 |
| *Pilea glauca* | 23 | p1 | (A)10 | 10 | 43591 | 43600 |
| *Pilea glauca* | 24 | p1 | (A)10 | 10 | 45347 | 45356 |
| *Pilea glauca* | 25 | p2 | (TA)5 | 10 | 46273 | 46282 |
| *Pilea glauca* | 26 | p4 | (TTTA)3 | 12 | 47956 | 47967 |
| *Pilea glauca* | 27 | p1 | (A)11 | 11 | 49829 | 49839 |
| *Pilea glauca* | 28 | p1 | (T)10 | 10 | 53619 | 53628 |
| *Pilea glauca* | 29 | p3 | (TTC)4 | 12 | 55448 | 55459 |
| *Pilea glauca* | 30 | p1 | (A)11 | 11 | 55978 | 55988 |
| *Pilea glauca* | 31 | p1 | (A)10 | 10 | 58535 | 58544 |
| *Pilea glauca* | 32 | p1 | (A)11 | 11 | 59351 | 59361 |
| *Pilea glauca* | 33 | p1 | (T)11 | 11 | 59648 | 59658 |
| *Pilea glauca* | 34 | p2 | (TC)5 | 10 | 60120 | 60129 |
| *Pilea glauca* | 35 | p1 | (A)15 | 15 | 63764 | 63778 |
| *Pilea glauca* | 36 | p2 | (TA)5 | 10 | 63974 | 63983 |
| *Pilea glauca* | 37 | p1 | (T)10 | 10 | 64616 | 64625 |
| *Pilea glauca* | 38 | p1 | (A)11 | 11 | 65180 | 65190 |
| *Pilea glauca* | 39 | p1 | (T)12 | 12 | 65903 | 65914 |
| *Pilea glauca* | 40 | p1 | (T)10 | 10 | 66864 | 66873 |
| *Pilea glauca* | 41 | p1 | (T)18 | 18 | 68996 | 69013 |
| *Pilea glauca* | 42 | p1 | (A)13 | 13 | 69665 | 69677 |
| *Pilea glauca* | 43 | p1 | (A)12 | 12 | 69701 | 69712 |
| *Pilea glauca* | 44 | p1 | (A)10 | 10 | 70451 | 70460 |
| *Pilea glauca* | 45 | p1 | (T)10 | 10 | 72487 | 72496 |
| *Pilea glauca* | 46 | p4 | (TTTC)3 | 12 | 73299 | 73310 |
| *Pilea glauca* | 47 | p1 | (T)10 | 10 | 74995 | 75004 |
| *Pilea glauca* | 48 | p5 | (AATTG)3 | 15 | 75374 | 75388 |
| *Pilea glauca* | 49 | p1 | (T)10 | 10 | 77381 | 77390 |
| *Pilea glauca* | 50 | p1 | (T)10 | 10 | 79509 | 79518 |
| *Pilea glauca* | 51 | p1 | (T)11 | 11 | 80683 | 80693 |
| *Pilea glauca* | 52 | p1 | (T)14 | 14 | 80807 | 80820 |
| *Pilea glauca* | 53 | p2 | (TA)6 | 12 | 91509 | 91520 |
| *Pilea glauca* | 54 | p1 | (A)21 | 21 | 105912 | 105932 |
| *Pilea glauca* | 55 | p1 | (T)11 | 11 | 108846 | 108856 |
| *Pilea glauca* | 56 | p4 | (ATAG)4 | 16 | 110042 | 110057 |
| *Pilea glauca* | 57 | p1 | (A)10 | 10 | 110343 | 110352 |
| *Pilea glauca* | 58 | p1 | (T)11 | 11 | 110640 | 110650 |
| *Pilea glauca* | 59 | p1 | (T)11 | 11 | 111312 | 111322 |
| *Pilea glauca* | 60 | p1 | (A)13 | 13 | 111663 | 111675 |
| *Pilea glauca* | 61 | p1 | (T)12 | 12 | 113065 | 113076 |
| *Pilea glauca* | 62 | p1 | (T)10 | 10 | 113448 | 113457 |
| *Pilea glauca* | 63 | p2 | (TA)5 | 10 | 118579 | 118588 |
| *Pilea glauca* | 64 | p1 | (T)10 | 10 | 123588 | 123597 |
| *Pilea glauca* | 65 | p1 | (T)13 | 13 | 124068 | 124080 |
| *Pilea glauca* | 66 | p1 | (T)10 | 10 | 125179 | 125188 |
| *Pilea glauca* | 67 | p1 | (T)21 | 21 | 127941 | 127961 |
| *Pilea glauca* | 68 | p2 | (TA)6 | 12 | 142353 | 142364 |
| *Pilea mollis* | 1 | p1 | (A)11 | 11 | 110 | 120 |
| *Pilea mollis* | 2 | p1 | (A)11 | 11 | 3608 | 3618 |
| *Pilea mollis* | 3 | p1 | (A)10 | 10 | 4380 | 4389 |
| *Pilea mollis* | 4 | p4 | (ATCA)3 | 12 | 4704 | 4715 |
| *Pilea mollis* | 5 | p1 | (A)11 | 11 | 5980 | 5990 |
| *Pilea mollis* | 6 | p1 | (A)11 | 11 | 7518 | 7528 |
| *Pilea mollis* | 7 | p1 | (A)15 | 15 | 8145 | 8159 |
| *Pilea mollis* | 8 | p1 | (A)11 | 11 | 8368 | 8378 |
| *Pilea mollis* | 9 | p1 | (T)11 | 11 | 12189 | 12199 |
| *Pilea mollis* | 10 | p3 | (TTA)5 | 15 | 15021 | 15035 |
| *Pilea mollis* | 11 | p1 | (T)12 | 12 | 15873 | 15884 |
| *Pilea mollis* | 12 | p1 | (T)10 | 10 | 17866 | 17875 |
| *Pilea mollis* | 13 | p1 | (T)14 | 14 | 17972 | 17985 |
| *Pilea mollis* | 14 | p2 | (TA)5 | 10 | 19366 | 19375 |
| *Pilea mollis* | 15 | p1 | (T)10 | 10 | 25738 | 25747 |
| *Pilea mollis* | 16 | p4 | (ATAG)3 | 12 | 27717 | 27728 |
| *Pilea mollis* | 17 | p4 | (ATAG)3 | 12 | 27738 | 27749 |
| *Pilea mollis* | 18 | p1 | (A)11 | 11 | 29547 | 29557 |
| *Pilea mollis* | 19 | p1 | (T)13 | 13 | 30429 | 30441 |
| *Pilea mollis* | 20 | p1 | (A)11 | 11 | 30544 | 30554 |
| *Pilea mollis* | 21 | p1 | (G)11 | 11 | 33384 | 33394 |
| *Pilea mollis* | 22 | p1 | (C)12 | 12 | 35611 | 35622 |
| *Pilea mollis* | 23 | p1 | (A)10 | 10 | 35623 | 35632 |
| *Pilea mollis* | 24 | p1 | (A)12 | 12 | 43383 | 43394 |
| *Pilea mollis* | 25 | p1 | (A)10 | 10 | 45132 | 45141 |
| *Pilea mollis* | 26 | p2 | (TA)5 | 10 | 45259 | 45268 |
| *Pilea mollis* | 27 | p4 | (TTTA)3 | 12 | 47764 | 47775 |
| *Pilea mollis* | 28 | p1 | (A)13 | 13 | 49640 | 49652 |
| *Pilea mollis* | 29 | p1 | (T)10 | 10 | 53089 | 53098 |
| *Pilea mollis* | 30 | p1 | (T)13 | 13 | 53476 | 53488 |
| *Pilea mollis* | 31 | p3 | (TTC)4 | 12 | 55308 | 55319 |
| *Pilea mollis* | 32 | p1 | (A)11 | 11 | 55839 | 55849 |
| *Pilea mollis* | 33 | p1 | (A)15 | 15 | 59244 | 59258 |
| *Pilea mollis* | 34 | p1 | (T)14 | 14 | 59551 | 59564 |
| *Pilea mollis* | 35 | p2 | (TC)5 | 10 | 60025 | 60034 |
| *Pilea mollis* | 36 | p1 | (A)13 | 13 | 63198 | 63210 |
| *Pilea mollis* | 37 | p2 | (TA)6 | 12 | 63407 | 63418 |
| *Pilea mollis* | 38 | p4 | (TTAA)3 | 12 | 64026 | 64037 |
| *Pilea mollis* | 39 | p1 | (T)11 | 11 | 64044 | 64054 |
| *Pilea mollis* | 40 | p1 | (T)10 | 10 | 67868 | 67877 |
| *Pilea mollis* | 41 | p1 | (T)13 | 13 | 68407 | 68419 |
| *Pilea mollis* | 42 | p1 | (A)11 | 11 | 68574 | 68584 |
| *Pilea mollis* | 43 | p1 | (A)11 | 11 | 69074 | 69084 |
| *Pilea mollis* | 44 | p1 | (A)10 | 10 | 69864 | 69873 |
| *Pilea mollis* | 45 | p4 | (TTTC)3 | 12 | 72704 | 72715 |
| *Pilea mollis* | 46 | p1 | (T)10 | 10 | 74969 | 74978 |
| *Pilea mollis* | 47 | p4 | (AATA)3 | 12 | 76031 | 76042 |
| *Pilea mollis* | 48 | p1 | (T)11 | 11 | 80207 | 80217 |
| *Pilea mollis* | 49 | p2 | (TA)6 | 12 | 90910 | 90921 |
| *Pilea mollis* | 50 | p1 | (A)11 | 11 | 104946 | 104956 |
| *Pilea mollis* | 51 | p1 | (A)12 | 12 | 105282 | 105293 |
| *Pilea mollis* | 52 | p6 | (AAAATT)3 | 18 | 106515 | 106532 |
| *Pilea mollis* | 53 | p1 | (A)10 | 10 | 108109 | 108118 |
| *Pilea mollis* | 54 | p1 | (T)11 | 11 | 108225 | 108235 |
| *Pilea mollis* | 55 | p4 | (ATAG)4 | 16 | 109421 | 109436 |
| *Pilea mollis* | 56 | p1 | (A)11 | 11 | 109642 | 109652 |
| *Pilea mollis* | 57 | p1 | (A)10 | 10 | 109726 | 109735 |
| *Pilea mollis* | 58 | p1 | (T)10 | 10 | 110022 | 110031 |
| *Pilea mollis* | 59 | p1 | (T)10 | 10 | 110695 | 110704 |
| *Pilea mollis* | 60 | p1 | (A)11 | 11 | 111044 | 111054 |
| *Pilea mollis* | 61 | p4 | (TAAA)3 | 12 | 111475 | 111486 |
| *Pilea mollis* | 62 | p1 | (T)12 | 12 | 112449 | 112460 |
| *Pilea mollis* | 63 | p1 | (T)11 | 11 | 112832 | 112842 |
| *Pilea mollis* | 64 | p1 | (A)10 | 10 | 114595 | 114604 |
| *Pilea mollis* | 65 | p1 | (T)10 | 10 | 118035 | 118044 |
| *Pilea mollis* | 66 | p1 | (T)10 | 10 | 120950 | 120959 |
| *Pilea mollis* | 67 | p1 | (T)10 | 10 | 122993 | 123002 |
| *Pilea mollis* | 68 | p1 | (T)13 | 13 | 123470 | 123482 |
| *Pilea mollis* | 69 | p3 | (TAC)4 | 12 | 124327 | 124338 |
| *Pilea mollis* | 70 | p1 | (T)10 | 10 | 124499 | 124508 |
| *Pilea mollis* | 71 | p1 | (T)10 | 10 | 124584 | 124593 |
| *Pilea mollis* | 72 | p6 | (TTTTAA)3 | 18 | 126115 | 126132 |
| *Pilea mollis* | 73 | p1 | (T)12 | 12 | 127358 | 127369 |
| *Pilea mollis* | 74 | p1 | (T)11 | 11 | 127695 | 127705 |
| *Pilea mollis* | 75 | p2 | (TA)6 | 12 | 141730 | 141741 |
| *Pilea peperomioides* | 1 | p1 | (A)10 | 10 | 3635 | 3644 |
| *Pilea peperomioides* | 2 | p4 | (ATCA)3 | 12 | 4704 | 4715 |
| *Pilea peperomioides* | 3 | p1 | (A)10 | 10 | 5972 | 5981 |
| *Pilea peperomioides* | 4 | p2 | (AT)7 | 14 | 6303 | 6316 |
| *Pilea peperomioides* | 5 | p2 | (TA)5 | 10 | 6318 | 6327 |
| *Pilea peperomioides* | 6 | p2 | (AT)6 | 12 | 6386 | 6397 |
| *Pilea peperomioides* | 7 | p1 | (A)10 | 10 | 6731 | 6740 |
| *Pilea peperomioides* | 8 | p3 | (TAT)4 | 12 | 6836 | 6847 |
| *Pilea peperomioides* | 9 | p3 | (ATA)4 | 12 | 6859 | 6870 |
| *Pilea peperomioides* | 10 | p1 | (A)12 | 12 | 7932 | 7943 |
| *Pilea peperomioides* | 11 | p2 | (TA)8 | 16 | 7844 | 7959 |
| *Pilea peperomioides* | 12 | p4 | (GTTT)3 | 12 | 8172 | 8183 |
| *Pilea peperomioides* | 13 | p1 | (T)10 | 10 | 8181 | 8190 |
| *Pilea peperomioides* | 14 | p1 | (A)17 | 17 | 8257 | 8273 |
| *Pilea peperomioides* | 15 | p1 | (A)13 | 13 | 8488 | 8500 |
| *Pilea peperomioides* | 16 | p3 | (TTA)5 | 15 | 15242 | 15256 |
| *Pilea peperomioides* | 17 | p1 | (T)12 | 12 | 15989 | 16000 |
| *Pilea peperomioides* | 18 | p1 | (T)10 | 10 | 18079 | 18088 |
| *Pilea peperomioides* | 19 | p1 | (T)14 | 14 | 18185 | 18198 |
| *Pilea peperomioides* | 20 | p2 | (TA)5 | 10 | 19573 | 19582 |
| *Pilea peperomioides* | 21 | p1 | (T)10 | 10 | 25911 | 25920 |
| *Pilea peperomioides* | 22 | p1 | (T)10 | 10 | 31465 | 31474 |
| *Pilea peperomioides* | 23 | p1 | (G)10 | 10 | 33619 | 33628 |
| *Pilea peperomioides* | 24 | p2 | (AT)5 | 10 | 34888 | 34897 |
| *Pilea peperomioides* | 25 | p1 | (C)10 | 10 | 35877 | 35886 |
| *Pilea peperomioides* | 26 | p1 | (A)10 | 10 | 35887 | 35896 |
| *Pilea peperomioides* | 27 | p1 | (A)11 | 11 | 46357 | 46367 |
| *Pilea peperomioides* | 28 | p2 | (AT)10 | 20 | 46547 | 46566 |
| *Pilea peperomioides* | 29 | p2 | (AT)5 | 10 | 46568 | 46577 |
| *Pilea peperomioides* | 30 | p1 | (T)10 | 10 | 53630 | 53639 |
| *Pilea peperomioides* | 31 | p1 | (A)12 | 12 | 58955 | 58966 |
| *Pilea peperomioides* | 32 | p2 | (AT)5 | 10 | 59038 | 59047 |
| *Pilea peperomioides* | 33 | p1 | (A)11 | 11 | 59780 | 59790 |
| *Pilea peperomioides* | 34 | p1 | (T)12 | 12 | 59942 | 59953 |
| *Pilea peperomioides* | 35 | p2 | (TC)5 | 10 | 60581 | 60590 |
| *Pilea peperomioides* | 36 | p1 | (A)11 | 11 | 64481 | 64491 |
| *Pilea peperomioides* | 37 | p1 | (A)11 | 11 | 64509 | 64519 |
| *Pilea peperomioides* | 38 | p1 | (T)11 | 11 | 64538 | 64548 |
| *Pilea peperomioides* | 39 | p1 | (T)12 | 12 | 65302 | 65313 |
| *Pilea peperomioides* | 40 | p1 | (T)10 | 10 | 65803 | 65812 |
| *Pilea peperomioides* | 41 | p1 | (T)10 | 10 | 65919 | 65928 |
| *Pilea peperomioides* | 42 | p4 | (AATT)3 | 12 | 66387 | 66398 |
| *Pilea peperomioides* | 43 | p1 | (A)10 | 10 | 67453 | 67462 |
| *Pilea peperomioides* | 44 | p1 | (T)15 | 15 | 69604 | 69618 |
| *Pilea peperomioides* | 45 | p2 | (AT)5 | 10 | 69636 | 69645 |
| *Pilea peperomioides* | 46 | p1 | (A)10 | 10 | 69775 | 69784 |
| *Pilea peperomioides* | 47 | p1 | (A)11 | 11 | 70277 | 70287 |
| *Pilea peperomioides* | 48 | p1 | (T)10 | 10 | 70309 | 70318 |
| *Pilea peperomioides* | 49 | p1 | (T)13 | 13 | 70537 | 70549 |
| *Pilea peperomioides* | 50 | p2 | (AT)9 | 18 | 73035 | 73052 |
| *Pilea peperomioides* | 51 | p1 | (T)11 | 11 | 78525 | 78535 |
| *Pilea peperomioides* | 52 | p1 | (A)11 | 11 | 80853 | 80863 |
| *Pilea peperomioides* | 53 | p1 | (T)16 | 16 | 81651 | 81666 |
| *Pilea peperomioides* | 54 | p2 | (TA)6 | 12 | 92163 | 92174 |
| *Pilea peperomioides* | 55 | p1 | (T)11 | 11 | 109453 | 109463 |
| *Pilea peperomioides* | 56 | p4 | (ATAG)4 | 16 | 110649 | 110664 |
| *Pilea peperomioides* | 57 | p1 | (T)10 | 10 | 111265 | 111274 |
| *Pilea peperomioides* | 58 | p5 | (TTTTA)3 | 15 | 112200 | 112214 |
| *Pilea peperomioides* | 59 | p3 | (TAT)4 | 12 | 112259 | 112270 |
| *Pilea peperomioides* | 60 | p1 | (T)10 | 10 | 114331 | 114340 |
| *Pilea peperomioides* | 61 | p4 | (ATTT)3 | 12 | 118543 | 118554 |
| *Pilea peperomioides* | 62 | p2 | (TA)5 | 10 | 119300 | 119309 |
| *Pilea peperomioides* | 63 | p1 | (A)11 | 11 | 123378 | 123388 |
| *Pilea peperomioides* | 64 | p1 | (A)10 | 10 | 123528 | 123537 |
| *Pilea peperomioides* | 65 | p1 | (A)10 | 10 | 124017 | 124026 |
| *Pilea peperomioides* | 66 | p1 | (T)10 | 10 | 124332 | 124341 |
| *Pilea peperomioides* | 67 | p1 | (T)13 | 13 | 124812 | 124824 |
| *Pilea peperomioides* | 68 | p1 | (T)10 | 10 | 124955 | 124964 |
| *Pilea peperomioides* | 69 | p1 | (T)12 | 12 | 126276 | 126287 |
| *Pilea peperomioides* | 70 | p1 | (T)10 | 10 | 126546 | 126555 |
| *Pilea peperomioides* | 71 | p2 | (TA)6 | 12 | 143446 | 143457 |
| *Pilea serpyllacea* | 1 | p1 | (A)10 | 10 | 104 | 113 |
| *Pilea serpyllacea* | 2 | p1 | (A)15 | 15 | 1482 | 1496 |
| *Pilea serpyllacea* | 3 | p1 | (T)11 | 11 | 4348 | 4358 |
| *Pilea serpyllacea* | 4 | p1 | (A)13 | 13 | 4362 | 4374 |
| *Pilea serpyllacea* | 5 | p1 | (A)10 | 10 | 4633 | 4642 |
| *Pilea serpyllacea* | 6 | p4 | (CAAT)3 | 12 | 4679 | 4690 |
| *Pilea serpyllacea* | 7 | p1 | (T)11 | 11 | 4729 | 4739 |
| *Pilea serpyllacea* | 8 | p4 | (TTAA)3 | 12 | 4939 | 4950 |
| *Pilea serpyllacea* | 9 | p1 | (A)14 | 14 | 8318 | 8331 |
| *Pilea serpyllacea* | 10 | p1 | (A)16 | 16 | 9631 | 9649 |
| *Pilea serpyllacea* | 11 | p2 | (AT)6 | 12 | 9741 | 9752 |
| *Pilea serpyllacea* | 12 | p1 | (T)13 | 13 | 12140 | 12152 |
| *Pilea serpyllacea* | 13 | p1 | (A)10 | 10 | 12308 | 12317 |
| *Pilea serpyllacea* | 14 | p3 | (TTA)4 | 12 | 14987 | 14998 |
| *Pilea serpyllacea* | 15 | p1 | (T)10 | 10 | 17795 | 17804 |
| *Pilea serpyllacea* | 16 | p1 | (T)14 | 14 | 17901 | 17914 |
| *Pilea serpyllacea* | 17 | p2 | (TA)5 | 10 | 19289 | 19298 |
| *Pilea serpyllacea* | 18 | p1 | (T)11 | 11 | 26735 | 26745 |
| *Pilea serpyllacea* | 19 | p4 | (ATAG)3 | 12 | 27638 | 27649 |
| *Pilea serpyllacea* | 20 | p4 | (TTAT)3 | 12 | 27807 | 27818 |
| *Pilea serpyllacea* | 21 | p1 | (T)10 | 10 | 28957 | 28966 |
| *Pilea serpyllacea* | 22 | p1 | (A)12 | 12 | 29454 | 29465 |
| *Pilea serpyllacea* | 23 | p1 | (T)12 | 12 | 30337 | 30348 |
| *Pilea serpyllacea* | 24 | p1 | (G)10 | 10 | 33383 | 33392 |
| *Pilea serpyllacea* | 25 | p1 | (A)10 | 10 | 35669 | 35678 |
| *Pilea serpyllacea* | 26 | p1 | (T)12 | 12 | 41813 | 41824 |
| *Pilea serpyllacea* | 27 | p1 | (A)13 | 13 | 43405 | 43417 |
| *Pilea serpyllacea* | 28 | p1 | (A)11 | 11 | 45109 | 45119 |
| *Pilea serpyllacea* | 29 | p4 | (AAAT)3 | 12 | 45131 | 45142 |
| *Pilea serpyllacea* | 30 | p4 | (TTTA)3 | 12 | 47654 | 47665 |
| *Pilea serpyllacea* | 31 | p1 | (A)10 | 10 | 50225 | 50234 |
| *Pilea serpyllacea* | 32 | p1 | (T)11 | 11 | 53122 | 53132 |
| *Pilea serpyllacea* | 33 | p1 | (T)10 | 10 | 53533 | 53542 |
| *Pilea serpyllacea* | 34 | p3 | (TTC)4 | 12 | 55350 | 55361 |
| *Pilea serpyllacea* | 35 | p1 | (A)12 | 12 | 55894 | 55905 |
| *Pilea serpyllacea* | 36 | p1 | (A)11 | 11 | 58439 | 58449 |
| *Pilea serpyllacea* | 37 | p1 | (A)13 | 13 | 59259 | 59271 |
| *Pilea serpyllacea* | 38 | p1 | (T)11 | 11 | 59563 | 59573 |
| *Pilea serpyllacea* | 39 | p2 | (TC)5 | 10 | 60034 | 60043 |
| *Pilea serpyllacea* | 40 | p1 | (T)10 | 10 | 62134 | 62143 |
| *Pilea serpyllacea* | 41 | p1 | (A)10 | 10 | 63684 | 63693 |
| *Pilea serpyllacea* | 42 | p2 | (TA)6 | 12 | 63883 | 63894 |
| *Pilea serpyllacea* | 43 | p1 | (A)11 | 11 | 64705 | 64715 |
| *Pilea serpyllacea* | 44 | p1 | (A)10 | 10 | 65133 | 65142 |
| *Pilea serpyllacea* | 45 | p1 | (T)13 | 13 | 66209 | 66221 |
| *Pilea serpyllacea* | 46 | p1 | (A)10 | 10 | 66649 | 66658 |
| *Pilea serpyllacea* | 47 | p5 | (TAAAT)3 | 15 | 67268 | 67282 |
| *Pilea serpyllacea* | 48 | p1 | (T)10 | 10 | 68870 | 68879 |
| *Pilea serpyllacea* | 49 | p2 | (AT)6 | 12 | 68897 | 68908 |
| *Pilea serpyllacea* | 50 | p1 | (A)16 | 16 | 69046 | 69061 |
| *Pilea serpyllacea* | 51 | p1 | (A)13 | 13 | 69551 | 69563 |
| *Pilea serpyllacea* | 52 | p1 | (A)12 | 12 | 69595 | 69606 |
| *Pilea serpyllacea* | 53 | p4 | (TTTA)4 | 16 | 72403 | 72418 |
| *Pilea serpyllacea* | 54 | p1 | (A)10 | 10 | 73732 | 73741 |
| *Pilea serpyllacea* | 55 | p1 | (T)10 | 10 | 77816 | 77825 |
| *Pilea serpyllacea* | 56 | p1 | (T)11 | 11 | 79919 | 79929 |
| *Pilea serpyllacea* | 57 | p1 | (T)11 | 11 | 80570 | 80580 |
| *Pilea serpyllacea* | 58 | p1 | (T)11 | 11 | 82681 | 82691 |
| *Pilea serpyllacea* | 59 | p1 | (T)13 | 13 | 85898 | 85910 |
| *Pilea serpyllacea* | 60 | p1 | (A)15 | 15 | 105634 | 105648 |
| *Pilea serpyllacea* | 61 | p1 | (T)11 | 11 | 108557 | 108567 |
| *Pilea serpyllacea* | 62 | p4 | (ATAG)4 | 16 | 109753 | 109768 |
| *Pilea serpyllacea* | 63 | p1 | (T)11 | 11 | 110350 | 110360 |
| *Pilea serpyllacea* | 64 | p1 | (A)14 | 14 | 110885 | 110898 |
| *Pilea serpyllacea* | 65 | p1 | (A)10 | 10 | 111236 | 111245 |
| *Pilea serpyllacea* | 66 | p1 | (T)12 | 12 | 112288 | 112299 |
| *Pilea serpyllacea* | 67 | p1 | (T)10 | 10 | 112745 | 112754 |
| *Pilea serpyllacea* | 68 | p1 | (A)10 | 10 | 115495 | 115504 |
| *Pilea serpyllacea* | 69 | p4 | (ATAA)3 | 12 | 116110 | 116121 |
| *Pilea serpyllacea* | 70 | p1 | (A)11 | 11 | 116157 | 116167 |
| *Pilea serpyllacea* | 71 | p1 | (T)12 | 12 | 117881 | 117892 |
| *Pilea serpyllacea* | 72 | p1 | (T)10 | 10 | 120800 | 120809 |
| *Pilea serpyllacea* | 73 | p1 | (A)10 | 10 | 122573 | 122582 |
| *Pilea serpyllacea* | 74 | p1 | (T)11 | 11 | 122878 | 122888 |
| *Pilea serpyllacea* | 75 | p1 | (T)13 | 13 | 123359 | 123371 |
| *Pilea serpyllacea* | 76 | p1 | (T)10 | 10 | 123418 | 123427 |
| *Pilea serpyllacea* | 77 | p1 | (T)10 | 10 | 124539 | 124548 |
| *Pilea serpyllacea* | 78 | p1 | (T)15 | 15 | 127302 | 127316 |
| *Pilea serpyllacea* | 79 | p1 | (A)13 | 13 | 147040 | 147052 |
| *Pilea serpyllacea* | 80 | p1 | (A)11 | 11 | 150259 | 150269 |

Note. We ignore the two adjacent SSRs to merge as a compound SSRs.
