# Supplementary material for: Comparative plastid genomics of four Pilea (Urticaceae) species: insight into interspecific plastid genome diversity in Pilea
Source: BMC Plant Biol. 2021 Jan 7;21:25. doi: 10.1186/s12870-020-02793-7 (PMC7792329; doi:10.1186/s12870-020-02793-7)
Supplement: Supplementary file 1 — Additional file 1: Table S1. Summary of sequencing data quality. Table S2. Gene composition in the plastid genomes of Pilea. Table S3. Statistics on simple sequence repeats (SSRs) in the 4 plastid genomes. Table S4. Repeats (> = 30 bp) identified in the four Pilea species. Table S5. Percentages of variable sites and Indels in orthologous genes among the 4 Pilea species. Table S6. The dS, dN and dN/dS values in 79 shared genes among 4 Pilea species. Table S7. List of plastid genomes used for phylogenetic analysis. Table S8. Summary information of the plant samples. [file 12870_2020_2793_MOESM1_ESM.zip › Table S5.docx]

**Table S5.** Percentages of variable sites and InDels in orthologous genes among the 4 *Pilea* species.

| No. | Gene | Variable sites | Indels | Aligned length (bp) | Mutation rates (%) |
| --- | --- | --- | --- | --- | --- |
| 1 | *ycf*1 | 941 | 35 | 5661 | 16.62 |
| 2 | *mat*K | 160 |  | 1518 | 10.54 |
| 3 | *ccs*A | 85 |  | 972 | 8.74 |
| 4 | *rps*15 | 23 |  | 273 | 8.42 |
| 5 | *ndh*F | 155 | 1 | 2268 | 6.83 |
| 6 | *rps*19 | 19 | 1 | 285 | 6.67 |
| 7 | *rpl*33 | 13 |  | 201 | 6.47 |
| 8 | *acc*D | 91 | 4 | 1422 | 6.40 |
| 9 | *rpl*22 | 21 |  | 333 | 6.31 |
| 10 | *clp*P | 37 |  | 591 | 6.26 |
| 11 | *rpo*C2 | 256 | 3 | 4188 | 6.11 |
| 12 | *ndh*G | 30 |  | 531 | 5.65 |
| 13 | *rps*16 | 15 | 1 | 270 | 5.56 |
| 14 | *psb*K | 10 |  | 186 | 5.38 |
| 15 | *rpo*A | 52 | 1 | 1026 | 5.07 |
| 16 | *cem*A | 34 |  | 696 | 4.89 |
| 17 | *rpl*16 | 18 |  | 369 | 4.88 |
| 18 | *ycf*4 | 27 |  | 555 | 4.86 |
| 19 | *rpl*20 | 17 | 1 | 360 | 4.72 |
| 20 | *ndh*A | 51 |  | 1092 | 4.67 |
| 21 | *ndh*I | 23 |  | 504 | 4.56 |
| 22 | *psa*J | 6 |  | 135 | 4.44 |
| 23 | *rps*8 | 18 |  | 405 | 4.44 |
| 24 | *pet*G | 5 |  | 114 | 4.39 |
| 25 | *psa*I | 5 |  | 114 | 4.39 |
| 26 | *rps*18 | 13 |  | 306 | 4.25 |
| 27 | *ndh*J | 20 |  | 477 | 4.19 |
| 28 | *pet*L | 4 |  | 96 | 4.17 |
| 29 | *atp*F | 23 |  | 555 | 4.14 |
| 30 | *psb*H | 9 |  | 222 | 4.05 |
| 31 | *ndh*D | 59 | 1 | 1530 | 3.86 |
| 32 | *rps*11 | 16 |  | 417 | 3.84 |
| 33 | *psb*M | 4 |  | 105 | 3.81 |
| 34 | *psb*N | 5 |  | 132 | 3.79 |
| 35 | *rpl*32 | 6 |  | 159 | 3.77 |
| 36 | *atp*A | 57 |  | 1524 | 3.74 |
| 37 | *pet*A | 36 |  | 963 | 3.74 |
| 38 | *rps*3 | 24 |  | 648 | 3.70 |
| 39 | *rpo*B | 116 |  | 3213 | 3.61 |
| 40 | *rpl*36 | 4 |  | 114 | 3.51 |
| 41 | *rpo*C1 | 71 | 1 | 2082 | 3.41 |
| 42 | *ndh*C | 12 |  | 363 | 3.31 |
| 43 | *rps*14 | 10 |  | 303 | 3.30 |
| 44 | *atp*E | 13 |  | 402 | 3.23 |
| 45 | *atp*I | 23 |  | 741 | 3.10 |
| 46 | *ndh*K | 20 |  | 681 | 2.94 |
| 47 | *atp*B | 43 |  | 1473 | 2.92 |
| 48 | *psa*C | 7 |  | 246 | 2.85 |
| 49 | *psb*B | 43 |  | 1527 | 2.82 |
| 50 | *rps*2 | 20 |  | 711 | 2.81 |
| 51 | *psb*I | 3 |  | 111 | 2.70 |
| 52 | *psa*B | 59 |  | 2205 | 2.68 |
| 53 | *ndh*H | 31 |  | 1182 | 2.62 |
| 54 | *psb*F | 3 |  | 120 | 2.50 |
| 55 | *pet*B | 16 |  | 648 | 2.47 |
| 56 | *rpl*2 | 10 |  | 408 | 2.45 |
| 57 | *psb*A | 25 |  | 1062 | 2.35 |
| 58 | *rps*4 | 14 |  | 606 | 2.31 |
| 59 | *psa*A | 52 |  | 2253 | 2.31 |
| 60 | *psb*D | 24 |  | 1062 | 2.26 |
| 61 | *pet*N | 2 |  | 90 | 2.22 |
| 62 | *psb*C | 31 |  | 1422 | 2.18 |
| 63 | *ycf*2 | 137 | 9 | 6849 | 2.00 |
| 64 | *ndh*E | 6 |  | 306 | 1.96 |
| 65 | *rbc*L | 28 |  | 1428 | 1.96 |
| 66 | *psb*T | 2 |  | 108 | 1.85 |
| 67 | *ycf*15 | 2 |  | 117 | 1.71 |
| 68 | *pet*D | 8 |  | 483 | 1.66 |
| 69 | *atp*H | 4 |  | 246 | 1.63 |
| 70 | *psb*E | 4 |  | 252 | 1.59 |
| 71 | *psb*Z | 3 |  | 189 | 1.59 |
| 72 | *ycf*3 | 8 |  | 507 | 1.58 |
| 73 | *rps*12 | 5 |  | 369 | 1.36 |
| 74 | *ndh*B | 15 |  | 1533 | 0.98 |
| 75 | *rps*7 | 4 |  | 468 | 0.85 |
| 76 | *rpl*14 | 7 |  | 825 | 0.85 |
| 77 | *rpl*23 | 2 |  | 282 | 0.71 |
| 78 | *psb*J | 0 |  | 123 | 0.00 |
| 79 | *psb*L | 0 |  | 198 | 0.00 |
