# Supplementary material for: Comparative plastid genomics of four Pilea (Urticaceae) species: insight into interspecific plastid genome diversity in Pilea
Source: BMC Plant Biol. 2021 Jan 7;21:25. doi: 10.1186/s12870-020-02793-7 (PMC7792329; doi:10.1186/s12870-020-02793-7)
Supplement: Supplementary file 1 — Additional file 1: Table S1. Summary of sequencing data quality. Table S2. Gene composition in the plastid genomes of Pilea. Table S3. Statistics on simple sequence repeats (SSRs) in the 4 plastid genomes. Table S4. Repeats (> = 30 bp) identified in the four Pilea species. Table S5. Percentages of variable sites and Indels in orthologous genes among the 4 Pilea species. Table S6. The dS, dN and dN/dS values in 79 shared genes among 4 Pilea species. Table S7. List of plastid genomes used for phylogenetic analysis. Table S8. Summary information of the plant samples. [file 12870_2020_2793_MOESM1_ESM.zip › Table S6.docx]

**Table S6.** The dS, dN and dN/dS values in 79 shared genes among 4 *Pilea* species.

| No. | Gene | dN | dS | dN/dS |
| --- | --- | --- | --- | --- |
| 1 | *ycf*2 | 0.023 | 0.0121 | 1.90343 |
| 2 | *rps*12 | 0.0149 | 0.0108 | 1.37779 |
| 3 | *pet*L | 0.0481 | 0.0375 | 1.28182 |
| 4 | *ycf*1 | 0.2253 | 0.1892 | 1.19077 |
| 5 | *rpl*36 | 0.0353 | 0.0407 | 0.86814 |
| 6 | *clp*P | 0.0724 | 0.0899 | 0.80566 |
| 7 | *acc*D | 0.068 | 0.0888 | 0.76569 |
| 8 | *mat*K | 0.1007 | 0.176 | 0.57241 |
| 9 | *rps*18 | 0.0418 | 0.0784 | 0.53254 |
| 10 | *ycf*15 | 0.0134 | 0.0271 | 0.49487 |
| 11 | *rpl*33 | 0.0572 | 0.1187 | 0.48134 |
| 12 | *rps*15 | 0.0763 | 0.1624 | 0.46957 |
| 13 | *ndh*G | 0.0469 | 0.1032 | 0.45445 |
| 14 | *rps*11 | 0.0313 | 0.0699 | 0.44751 |
| 15 | *psa*I | 0.0365 | 0.083 | 0.44036 |
| 16 | *ccs*A | 0.0771 | 0.1837 | 0.41968 |
| 17 | *rpo*C2 | 0.0506 | 0.1236 | 0.40885 |
| 18 | *ndh*B | 0.0071 | 0.0184 | 0.3874 |
| 19 | *rps*2 | 0.0226 | 0.0612 | 0.36847 |
| 20 | *psb*E | 0.0112 | 0.0312 | 0.35903 |
| 21 | *ndh*I | 0.0354 | 0.0987 | 0.35869 |
| 22 | *atp*F | 0.0338 | 0.095 | 0.35599 |
| 23 | *rpl*2 | 0.0186 | 0.0526 | 0.35417 |
| 24 | *cem*A | 0.0383 | 0.1137 | 0.33686 |
| 25 | *rps*19 | 0.0534 | 0.159 | 0.33604 |
| 26 | *rpl*20 | 0.0369 | 0.111 | 0.33281 |
| 27 | *rpl*22 | 0.0467 | 0.1414 | 0.33023 |
| 28 | *psb*K | 0.039 | 0.1187 | 0.32885 |
| 29 | *rps*14 | 0.0217 | 0.0786 | 0.27641 |
| 30 | *ndh*F | 0.0508 | 0.1997 | 0.25434 |
| 31 | *ndh*K | 0.0188 | 0.0757 | 0.24851 |
| 32 | *rpo*A | 0.0342 | 0.1383 | 0.24723 |
| 33 | *ndh*C | 0.0214 | 0.0896 | 0.23915 |
| 34 | *ndh*D | 0.0241 | 0.1032 | 0.2339 |
| 35 | *rpo*B | 0.0231 | 0.1001 | 0.23096 |
| 36 | *psb*H | 0.0261 | 0.1169 | 0.22363 |
| 37 | *rps*3 | 0.0245 | 0.1102 | 0.22277 |
| 38 | *rps*16 | 0.0489 | 0.222 | 0.22036 |
| 39 | *ycf*4 | 0.0308 | 0.1441 | 0.21353 |
| 40 | *rps*7 | 0.0053 | 0.0255 | 0.20671 |
| 41 | *ndh*A | 0.0289 | 0.144 | 0.20078 |
| 42 | *atp*I | 0.0176 | 0.0942 | 0.18728 |
| 43 | *rpo*C1 | 0.02 | 0.1081 | 0.18515 |
| 44 | *ndh*J | 0.0243 | 0.1324 | 0.18359 |
| 45 | *rps*4 | 0.0144 | 0.0784 | 0.18305 |
| 46 | *pet*A | 0.0183 | 0.1037 | 0.17648 |
| 47 | *rps*8 | 0.0226 | 0.136 | 0.16655 |
| 48 | *atp*E | 0.0161 | 0.1008 | 0.15941 |
| 49 | *rpl*16 | 0.0288 | 0.1832 | 0.1572 |
| 50 | *atp*A | 0.018 | 0.1162 | 0.15463 |
| 51 | *rbc*L | 0.0097 | 0.0637 | 0.15264 |
| 52 | *ndh*H | 0.0128 | 0.0895 | 0.14293 |
| 53 | *rpl*14 | 0.0033 | 0.0238 | 0.13876 |
| 54 | *psa*A | 0.0099 | 0.0727 | 0.13613 |
| 55 | *psb*Z | 0.007 | 0.0536 | 0.13111 |
| 56 | *ndh*E | 0.0083 | 0.0741 | 0.11225 |
| 57 | *ycf*3 | 0.0071 | 0.0647 | 0.11029 |
| 58 | *atp*B | 0.0108 | 0.107 | 0.10077 |
| 59 | *psb*N | 0.0113 | 0.1128 | 0.10044 |
| 60 | *psb*M | 0.0128 | 0.143 | 0.08951 |
| 61 | *pet*G | 0.0119 | 0.1348 | 0.0886 |
| 62 | *psa*C | 0.0103 | 0.116 | 0.08837 |
| 63 | *rpl*32 | 0.0155 | 0.2101 | 0.07375 |
| 64 | *psb*D | 0.0062 | 0.0856 | 0.07215 |
| 65 | *psb*C | 0.0049 | 0.0776 | 0.06258 |
| 66 | *psa*B | 0.0065 | 0.1114 | 0.05818 |
| 67 | *psa*J | 0.0106 | 0.1972 | 0.05354 |
| 68 | *pet*B | 0.004 | 0.1116 | 0.03553 |
| 69 | *psb*B | 0.0043 | 0.1229 | 0.03489 |
| 70 | *pet*D | 0.0028 | 0.0858 | 0.03213 |
| 71 | *psb*A | 0.0024 | 0.1107 | 0.02184 |
| 72 | *rpl*23 | 0.0096 | 0 | / |
| 73 | *atp*H | 0 | 0.073 | 0 |
| 74 | *pet*N | 0 | 0.0913 | 0 |
| 75 | *psb*F | 0 | 0.1001 | 0 |
| 76 | *psb*I | 0 | 0.1038 | 0 |
| 77 | *psb*J | 0 | 0 | / |
| 78 | *psb*L | 0 | 0 | / |
| 79 | *psb*T | 0 | 0.0841 | 0 |

Note. dS: synonymous substitution rates, dN: nonsynonymous substitution rates.
