# Supplementary material for: Comparative plastid genomics of four Pilea (Urticaceae) species: insight into interspecific plastid genome diversity in Pilea
Source: BMC Plant Biol. 2021 Jan 7;21:25. doi: 10.1186/s12870-020-02793-7 (PMC7792329; doi:10.1186/s12870-020-02793-7)
Supplement: Supplementary file 1 — Additional file 1: Table S1. Summary of sequencing data quality. Table S2. Gene composition in the plastid genomes of Pilea. Table S3. Statistics on simple sequence repeats (SSRs) in the 4 plastid genomes. Table S4. Repeats (> = 30 bp) identified in the four Pilea species. Table S5. Percentages of variable sites and Indels in orthologous genes among the 4 Pilea species. Table S6. The dS, dN and dN/dS values in 79 shared genes among 4 Pilea species. Table S7. List of plastid genomes used for phylogenetic analysis. Table S8. Summary information of the plant samples. [file 12870_2020_2793_MOESM1_ESM.zip › Table S7.docx]

**Table S7.** List of plastid genomes used for phylogenetic analysis.

| NO. | Species | Family | Subfamily | Accession number |
| --- | --- | --- | --- | --- |
| 1 | *Debregeasia elliptica* | Urticaceae | Boehmerioideae | MN189948.1 |
| 2 | *Debregeasia longifolia* | Urticaceae | Boehmerioideae | MN189952.1 |
| 3 | *Debregeasia orientalis* | Urticaceae | Boehmerioideae | MN189956.1 |
| 4 | *Debregeasia saeneb* | Urticaceae | Boehmerioideae | MN189958.1 |
| 5 | *Debregeasia squamata* | Urticaceae | Boehmerioideae | MN189959.1 |
| 6 | *Boehmeria nivea var. nipononivea* | Urticaceae | Boehmerioideae | MN189944.1 |
| 7 | *Boehmeria tomentosa* | Urticaceae | Boehmerioideae | MN189945.1 |
| 8 | *Rousselia humilis* | Urticaceae | Boehmerioideae | MN189969.1 |
| 9 | *Gonostegia hirta* | Urticaceae | Boehmerioideae | MN189962.1 |
| 10 | *Hemistylus odontophylla* | Urticaceae | Boehmerioideae | MN189963.1 |
| 11 | *Pipturus arborescens* | Urticaceae | Boehmerioideae | MN189967.1 |
| 12 | *Pouzolzia elegans var. elegans* | Urticaceae | Boehmerioideae | MN189968.1 |
| 13 | *Droguetia iners* | Urticaceae | Boehmerioideae | MN189960.1 |
| 14 | *Oreocnide frutescens* | Urticaceae | Boehmerioideae | MN189965.1 |
| 15 | *Parietaria micrantha* | Urticaceae | Boehmerioideae | MN189966.1 |
| 16 | *Cecropia pachystachya* | Urticaceae | Cecropioideae | NC_039763.1 |
| 17 | *Elatostema dissectum* | Urticaceae | Lecanthoideae | NC_047192.1 |
| 18 | *Elatostema laevissimum var. laevissimum* | Urticaceae | Lecanthoideae | MN189961.1 |
| 19 | *Pilea mollis* | Urticaceae | Lecanthoideae | MT726018 |
| 20 | *Pilea glauca* | Urticaceae | Lecanthoideae | MT726015 |
| 21 | *Pilea serpyllacea* | Urticaceae | Lecanthoideae | MT726017 |
| 22 | *Pilea peperomioides* | Urticaceae | Lecanthoideae | MT726016 |
| 23 | *Hesperocnide tenella* | Urticaceae | Urticoideae | MN189964.1 |
| 24 | *Morus indica* | Moraceae |  | NC_008359.1 |
| 25 | *Ficus carica* | Moraceae |  | NC_035237.1 |
