# Supplementary material for: Comparative plastid genomics of four Pilea (Urticaceae) species: insight into interspecific plastid genome diversity in Pilea
Source: BMC Plant Biol. 2021 Jan 7;21:25. doi: 10.1186/s12870-020-02793-7 (PMC7792329; doi:10.1186/s12870-020-02793-7)
Supplement: Supplementary file 1 — Additional file 1: Table S1. Summary of sequencing data quality. Table S2. Gene composition in the plastid genomes of Pilea. Table S3. Statistics on simple sequence repeats (SSRs) in the 4 plastid genomes. Table S4. Repeats (> = 30 bp) identified in the four Pilea species. Table S5. Percentages of variable sites and Indels in orthologous genes among the 4 Pilea species. Table S6. The dS, dN and dN/dS values in 79 shared genes among 4 Pilea species. Table S7. List of plastid genomes used for phylogenetic analysis. Table S8. Summary information of the plant samples. [file 12870_2020_2793_MOESM1_ESM.zip › Table S8.docx]

**Table S8.** Summary information of the plant samples.

| Sample | Collection places | Geospatial coordinate | Voucher code | Storage places |
| --- | --- | --- | --- | --- |
| *P*. *glauca* | Flower Expo Garden of Guangzhou | N23.0623/E113.2046 | UP200602 | Herbarium of Southwest university, Chongqing (No.2, Tiansheng Road, Beibei District, Chongqing, China) |
| *P*. *peperomioides* | Dounan Flowers Market, Chenggong District, Kunming | N24.9021/E102.7878 | UP200605 |  |
| *P*. *serpyllacea* | Flowers and Plants Market, Shuyang city, Jiangsu | N23.0480/E113.2224 | UP200603 |  |
| *P*. *mollis* | Baihui Horticultural Base, Guangzhou | N23.0623/E113.2046 | UP200604 |  |
