# Supplementary material for: Comparative plastid genomics of four Pilea (Urticaceae) species: insight into interspecific plastid genome diversity in Pilea
Source: BMC Plant Biol. 2021 Jan 7;21:25. doi: 10.1186/s12870-020-02793-7 (PMC7792329; doi:10.1186/s12870-020-02793-7)
Supplement: Supplementary file 1 — Additional file 1: Table S1. Summary of sequencing data quality. Table S2. Gene composition in the plastid genomes of Pilea. Table S3. Statistics on simple sequence repeats (SSRs) in the 4 plastid genomes. Table S4. Repeats (> = 30 bp) identified in the four Pilea species. Table S5. Percentages of variable sites and Indels in orthologous genes among the 4 Pilea species. Table S6. The dS, dN and dN/dS values in 79 shared genes among 4 Pilea species. Table S7. List of plastid genomes used for phylogenetic analysis. Table S8. Summary information of the plant samples. [file 12870_2020_2793_MOESM1_ESM.zip › Table.docx]

**Table 1.** Basic features of the 4 plastid genomes from *Pilea*.

| Species | | *P. glauca* | *P. mollis* | *P. peperomioides* | *P. serpyllacea* |
| --- | --- | --- | --- | --- | --- |
| Accession number | | MT726015 | MT726018 | MT726016 | MT726017 |
| Length (bp) | Total | 151,210 | 150,587 | 152,327 | 150,398 |
|  | LSC | 82,662 | 82,063 | 83,292 | 82,551 |
|  | SSC | 17,836 | 17,864 | 18,363 | 17,487 |
|  | IR | 25,356 | 25,330 | 25,336 | 25,180 |
| GC content (%) | Total | 36.69 | 36.72 | 36.35 | 36.41 |
|  | LSC | 34.31 | 34.36 | 33.87 | 33.96 |
|  | IR | 42.64 | 42.65 | 42.73 | 42.56 |
|  | SSC | 30.81 | 30.76 | 30.01 | 30.23 |
| Gene numbers | Total | 133 | 133 | 133 | 133 |
|  | Protein-coding gene | 88 | 88 | 88 | 88 |
|  | tRNA gene | 37 | 37 | 37 | 37 |
|  | rRNA gene | 8 | 8 | 8 | 8 |
